# Ammonium tetrathiomolybdate relieves oxidative stress in cisplatin-induced acute kidney injury via NRF2 signaling pathway

**DOI:** 10.1038/s41420-023-01564-1

**Published:** 2023-07-25

**Authors:** Hao Qi, Haoyu Shi, Minbo Yan, Liangyu Zhao, Yinghao Yin, Xiaolin Tan, Huiyue Qi, Hu Li, Kangqiang Weng, Yuxin Tang, Yingbo Dai

**Affiliations:** 1 Department of Urology, The Fifth Affiliated Hospital of Sun Yat-Sen University, Zhuhai, China; 2 Guangdong Provincial Key Laboratory of Biomedical Imaging, The Fifth Affiliated Hospital of Sun Yat-Sen University, Zhuhai, China; 3 Department of Clinical Nutrition, The Fifth Affiliated Hospital of Sun Yat-Sen University, Zhuhai, China

**Keywords:** Preclinical research, Ubiquitylation

## Abstract

Cisplatin is an efficient chemotherapeutic agent for various solid tumors, but its usage is restricted by nephrotoxicity. A single dose of cisplatin can cause acute kidney injury (AKI), which is characterized by rapid reduction in kidney function. However, the current therapies, such as hydration, are limited. It is vital to develop novel therapeutic reagents that have both anticancer and renoprotective properties. The objective of this study was to determine whether ammonium tetrathiomolybdate (TM), a copper chelator used to treat cancer and disorders of copper metabolism, may offer protection against cisplatin-induced AKI. In this study, we demonstrated that TM treatment had antioxidative effects and mitigated cisplatin-induced AKI both in vivo and in vitro. Mechanically, TM inhibited NRF2 ubiquitination, which activated the NRF2 pathway in HK-2 cells and promoted the expression of target genes. It should be noted that the protective effect conferred by TM against cisplatin was compromised by the knockdown of the NRF2 gene. Furthermore, TM selectively activated the NRF2 pathways in the liver and kidney. The current study provided evidence for additional clinical applications of TM by showing that it activates NRF2 and has a favorable therapeutic impact on cisplatin-induced AKI.

## Introduction

The kidneys maintain homeostasis by removing metabolic waste products as well as drugs and poisons, hence they are prone to injury induced by nephrotoxic drugs. Cisplatin is one of these medications that is quite effective in targeting rapidly dividing cells, but its clinical use is restricted by its nephrotoxicity [1]. Acute kidney injury (AKI) caused by cisplatin is clinically diagnosed by elevated serum creatinine blood levels, and treatment is limited. Furthermore, persistent kidney damage is seen notably even years after discontinuation of the drug. Even with existing approaches, 21.4% of patients still develop AKI [2]. A long-term study has confirmed that 30% of patients have abnormal renal function or hypertension after cisplatin treatment [3]. Most people report slight but persistent reductions in eGFR even in the absence of obvious symptoms [4]. Despite the fact that nephrotoxicity has been a problem for many years, the underlying mechanism is largely unknown. The toxic effect is initiated by the absorption into proximal tubular epithelial cells [5]. Then cisplatin causes DNA damage, leading to the accumulation of reactive oxygen species (ROS), which in turn triggers a severe inflammatory response and apoptosis [6]. As a result, oxidative stress is a significant contributor to the development of AKI and a powerful therapeutic target. But no medicine has yet received FDA approval for use in clinical practice.

The well-known master regulator of redox homeostasis, nuclear factor erythroid 2-related factor 2 (NRF2; encoded by NFE2L2), emerges as a significant mediator of the internal environment [7]. And several inducers of NRF2 have been characterized and studied with respect to the prevention and treatment of kidney diseases. For example, bardoxolone methyl [8], oltipraz [9], and sulforaphane [10] have shown great therapeutic potential in cisplatin-induced AKI. However, there are still no FDA-approved NRF2 inducers available to treat kidney diseases in humans. Therefore, more research is needed to increase the efficacy and specificity of NRF2 inducers.

Ammonium tetrathiomolybdate (TM), which is made up of a small inorganic tetrahedral molybdenum-sulfur anion (MoS_4_^2-^), is an anti-copper drug used to treat the neurologic presentation of Wilson’s disease. Since lowering copper levels can inhibit plenty of copper-dependent angiogenic cytokines, TM has shown excellent efficacy in the treatment of a variety of tumors, such as breast cancer [11]. Recently, studies have demonstrated that TM exhibits cyto- and organ- protective abilities against oxidative damage. Consequently, TM has been reported to alleviate skin inflammation [12], Alzheimer’s disease [13], heart ischemia‒reperfusion injury [14], and pulmonary fibrosis [15]. However, the protective function of TM in cisplatin-induced AKI has not been elucidated so far.

In the present study, we demonstrated that TM could alleviate cisplatin-induced AKI by exerting anti-oxidative effect both in vivo and in vitro. Mechanically, TM activated the NRF2 pathway in renal tubular epithelial cells and protected against cisplatin-induced AKI in a NRF2-dependent manner. Our findings reveal novel pharmacological applications of TM, which may provide a new therapeutic strategy for the treatment of cisplatin-induced AKI.

## Results

### TM alleviates cisplatin-induced cell damage in HK-2 cells

**Fig. 1:**
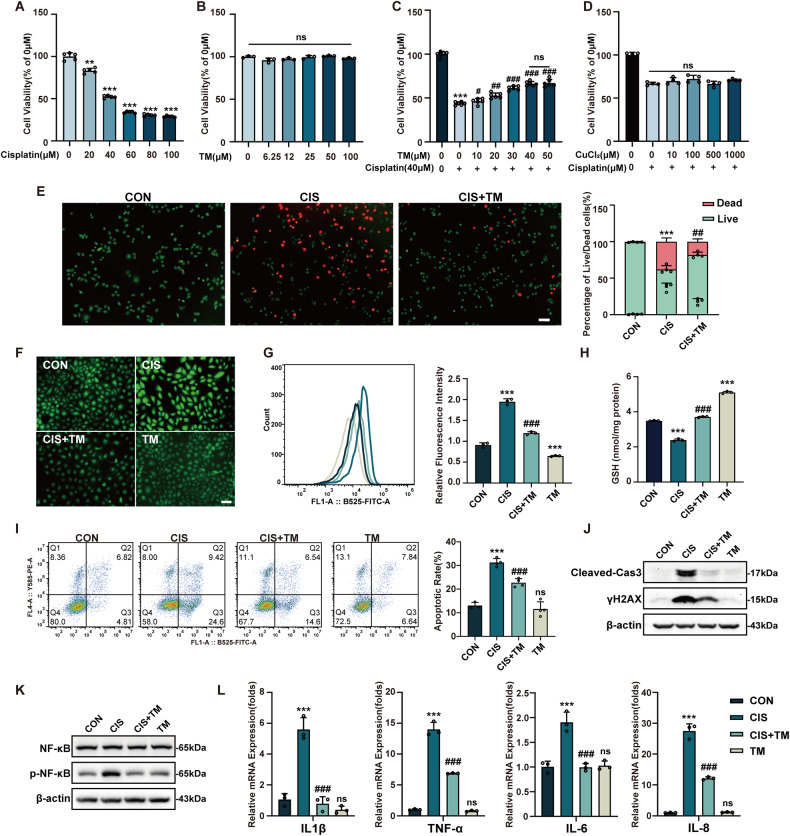
TM prevents the death of HK-2 cells caused by cisplatin by reducing oxidative stress, apoptosis, and inflammation. **A** Cytotoxicity of cisplatin is dose dependent. IC50 (40 μM) was used for the following experiments (n = 4). **B** Cell viability of HK-2 cells after TM treatment for 24 h (n = 3). **C** Treatment with TM blocked the cytotoxic effect of cisplatin(n = 4). **D** Copper ions did not make HK-2 cells more susceptible to cisplatin-induced death (n = 3). **E** HK-2 cells were stained with LIVE/DEAD^®^ Cell Imaging Kit. Viable cells(green) and dead cells(red) were represented and statistic analysis is on the right. Scale bars:100 μm. Fluorescence (**F**) and flow cytometry (**G**) detected the intracellular ROS level with CM-H2DCFDA(n = 3). Scale bars: 100 μm. **H** GSH levels of HK-2 cells (n = 3). **I** TM reduced the apoptotic rate in HK-2 cells (n = 3). **J**, **K** Western blot analysis of cleaved-caspase3, γH2AX, NF-κB and phosphorylated NF-κB levels in HK-2 cells. (**L**) Quantitative real-time PCR analysis of IL-1β, TNF-α, IL-6 and IL-8 (n = 3). ns: not significant. * p < 0.05, ** p < 0.01, *** p < 0.001 vs control group; *#p* < 0.05, *##p* < 0.01, *###p* < 0.001 vs cisplatin group. CON: control; CIS: cisplatin; TM: Ammonium tetrathiomolybdate. Data were presented as mean ± SD.

To explore the effect of TM against cell damage caused by cisplatin, the immortalized renal tubular epithelial cell (HK-2 cells) was used to establish a model of cisplatin nephrotoxicity in vitro. Cytotoxicity was dose-dependent (Fig. 1A) and cisplatin significantly induced death characterized by shrinkage, bubbling, and fragmentation of cells (Fig. S1A). And TM did not have toxic effects (Fig. 1B). Importantly, TM attenuated cisplatin cytotoxicity in a concentration-dependent manner (Fig. 1C). Furthermore, the morphological alteration was preserved after TM therapy. Live/dead staining showed that dead cells were significantly reduced after TM treatment (Fig. 1E). Since the main pharmacological effect of TM is copper chelation [16], the cytotoxic effect of copper ions was investigated. However, copper chloride did not increase cisplatin toxicity (Figs. S1B, Fig. 1D), indicating other potential effects in addition to copper chelation. A previous study identified TM as a novel H_2_S donor [17]. Subsequently, we revealed that TM released H_2_S in a dose-dependent way (Fig. S1C). However, there was no direct protective effect of H_2_S against cisplatin (Fig. S1D). In addition, referring to the Xue-xia Yuan research [18], we found that TM and cisplatin could not chelate to form precipitation (Fig. S1E, F).

Cisplatin has been reported to induce oxidative stress, apoptosis, and inflammation in the kidney [19]. In our study, cisplatin triggered robust ROS production, while co-incubation with TM suppressed ROS production (Fig. 1F, G). Glutathione (GSH), the principal reducing substance in cells, decreased after exposure to cisplatin and was rescued by TM treatment (Fig. 1H). Apoptosis can be evoked by oxidative stress [20]. Flow cytometry confirmed that cisplatin caused a significant increase in apoptosis rate, which was inhibited by TM (Fig. 1I). Accordingly, TM dampened the elevation of various apoptosis markers, such as cleaved-caspase3 and γ-H2AX compared to the cisplatin group (Fig. 1J). Finally, the nuclear factor kappa B (NF-κB), which plays a vital role in the inflammatory response [21], was also investigated. The phosphorylation of NF-κB was inhibited by TM compared to the cisplatin group (Fig. 1K). And inflammatory factors, such as IL-1β, TNF-α, IL-6, and IL-8, were alleviated by TM treatment (Fig. 1L). Collectively, these data verified the antioxidative, antiapoptotic and anti-inflammatory properties of TM in vitro.

### TM activates the NRF2 signaling pathway both in vitro and in vivo

**Fig. 2:**
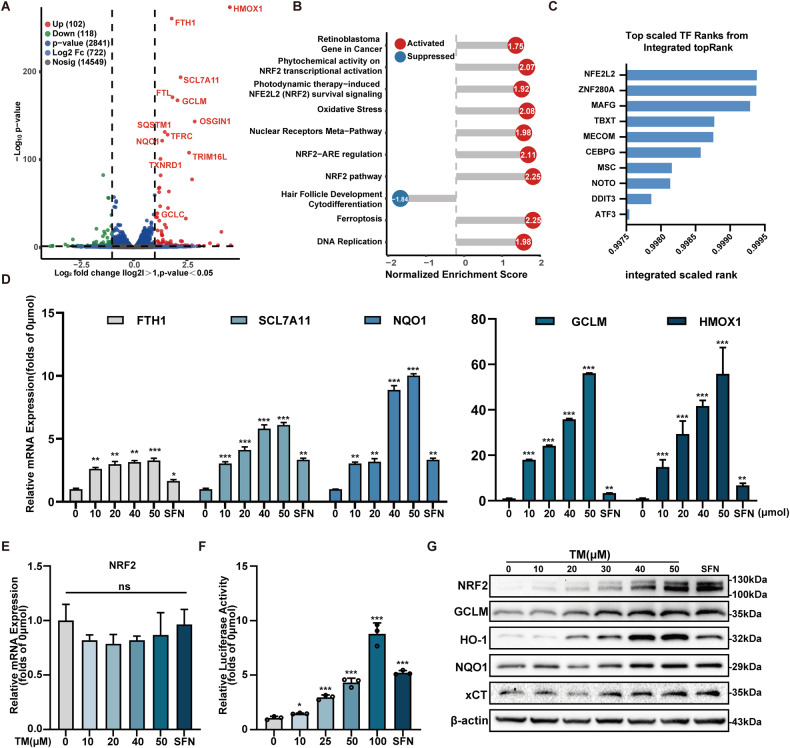
TM activates the NRF2 signaling pathway in vitro. **A** Volcano diagram of DEGs between the control and TM group, the genes highlighted in red are representative target genes of the NRF2 pathway. **B** GSEA enrichment revealed that genes related to the NRF2 pathway were activated. **C** ChEA3 orthogonal omics integration analysis of transcription factor. **D** TM treatment promoted the expression of *FTH1*, *SLC7A11*, *NQO1*, *GCLM* and *HMOX1* in a dose-dependent manner. SFN (Sulforaphane,20 μM) served as a positive control (n = 3). **E** The mRNA level of *NRF2* after TM treatment (n = 3). **F** ARE binding activity after treatment for 12 h (n = 3). **G** Representative Western blot of the proteins in vitro after TM treatment. SFN served as a positive control. ns: not significant. * p < 0.05, ** p < 0.01, *** p < 0.001 vs control group; Data were presented as mean ± SD.

To investigate the underlying mechanism of the protective effect of TM, transcriptome sequencing was applied. About 220 differentially expressed genes were found, 102 of these genes were up-regulated and 118 were down-regulated (Figs. S2A, Fig. 2A). KEGG analysis highlighted genes enriched in ferroptosis biosynthesis of cofactors, glutathione metabolism and mineral absorption (Fig. S2B). The GSEA enrichment strongly indicated that NRF2 signaling was involved (Fig. 2B). Furthermore, we predicted the transcription factor (TF) for gene signature by the ChEA3 (https://amp.pharm.mssm.edu/chea3/) [22]. The top 10 TFs were listed (Fig. 2C). NFE2L2, which encodes NRF2, was predicted to be the most closely related. The protein-protein interaction (PPI) network was constructed and visualized by STRING (Fig. S2C).

The mRNA expression patterns of NRF2 and five downstream genes, such as FTH1, SCL7A11, NQO1, GCLM, and HMOX1 were examined to further validate the sequencing results. As expected, TM increased downstream genes in a dose-dependent manner (Fig. 2D). However, the mRNA level of NRF2 was not elevated (Fig. 2E). The antioxidant response element (ARE) is found in the promoter region of several genes encoding detoxification enzymes and cytoprotective proteins, which is the target sequence of NRF2. After TM treatment, we detected a concentration-dependent increasing of ARE inductivity (Fig. 2F). Furthermore, TM treatment also increased protein levels of these genes in HK-2 cells (Fig. 2G). Additionally, the animal experiment showed great consistency in vitro. TM induced NRF2 and downstream gene expression in C57BL/6 mice kidneys (Fig. S3A, B). To our surprise, TM activated the NRF2 pathway in the kidney and liver without affecting the brain, heart, or lung (Fig. S3C). Taken together, these results demonstrate that TM is a potent NRF2 activator both in vitro and in vivo.

### NRF2 pathway activation confers TM protective effect against cisplatin-induced oxidative stress and apoptosis

**Fig. 3:**
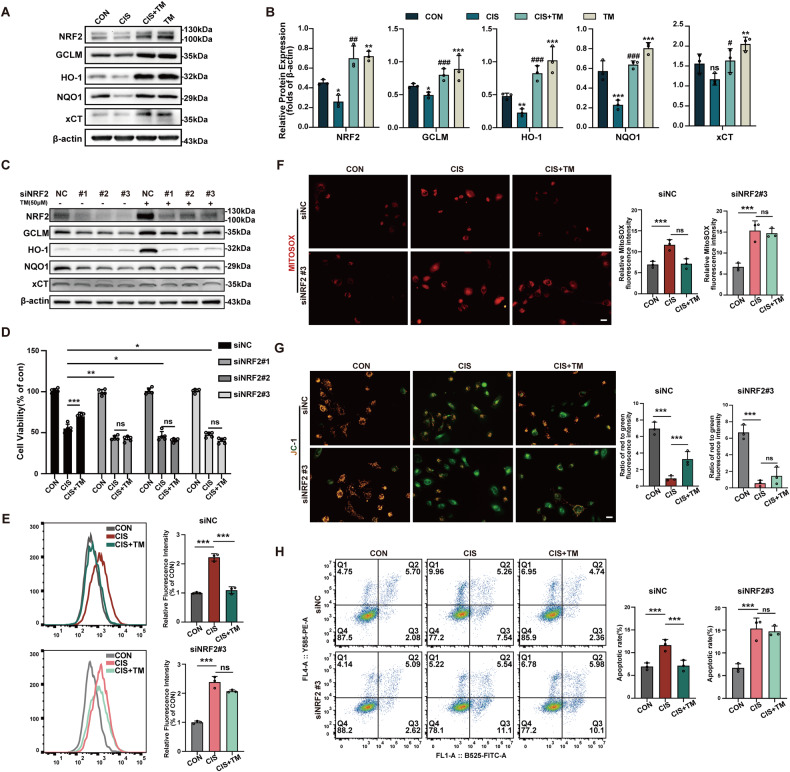
The protective effect of TM on HK-2 cells against cisplatin is dependent on NRF2. **A**, **B** Protein levels of NRF2, GCLM, HO-1, NQO1 and xCT were detected by Western blot after cisplatin treatment for 24 h (n = 3). **C** The NRF2 knockdown efficiency was determined. The siNRF2#3 was selected for following experiments. **D** The cell viability assay showed that NRF2 knockdown abolished the protective effect of TM against cisplatin (n = 4). The total ROS (**E**) and mitochondrial ROS (**F**) level in HK-2 cells transfected with siRNA was measured (n = 3). **G** JC-1 stain of control cells and siRNA#3 transfected cells after cisplatin treatment. **H** Analysis of the apoptotic rate of control cells and siRNA#3 transfected cells after cisplatin treatment using flow cytometry (n = 3). ns: not significant. * p < 0.05, ** p < 0.01, *** p < 0.001, vs. control group; *#p* < 0.05, *##p* < 0.01, *###p* < 0.001, vs. cisplatin group. CON: control; CIS: cisplatin; TM: Ammonium tetrathiomolybdate. Data were presented as the mean ± SD.

NRF2 has been widely established to play a protective role in cisplatin-induced AKI [23]. After 24 h of treatment, we observed that cisplatin(40μmol) inhibited NRF2 and several downstream genes in HK-2 cells, which were significantly promoted by TM (50μmol) (Fig. 3A, B). To interrogate if the TM-mediated protective effect was NRF2-dependent, HK-2 cells were transfected with siRNAs against human NRF2 before being treated (Fig. 3C). Subsequently, we found that the protective effect against cisplatin of TM was significantly weakened in NRF2-knockdown cells (Fig. 3D). As expected, total ROS and mitochondrial ROS induced by cisplatin was boosted with NRF2 knockdown, and NRF2 depletion abolished the effect of TM on ROS generation (Fig. 3E, F). Consistently, the protective effect of TM on NRF2 knockdown cells was nearly weakened in terms of apoptosis (Fig. 3G, H). Taken together, these results suggest that NRF2 mitigates the cytoprotective effect of TM.

### TM inhibits NRF2 ubiquitination and promotes its stability and nuclear translocation

**Fig. 4:**
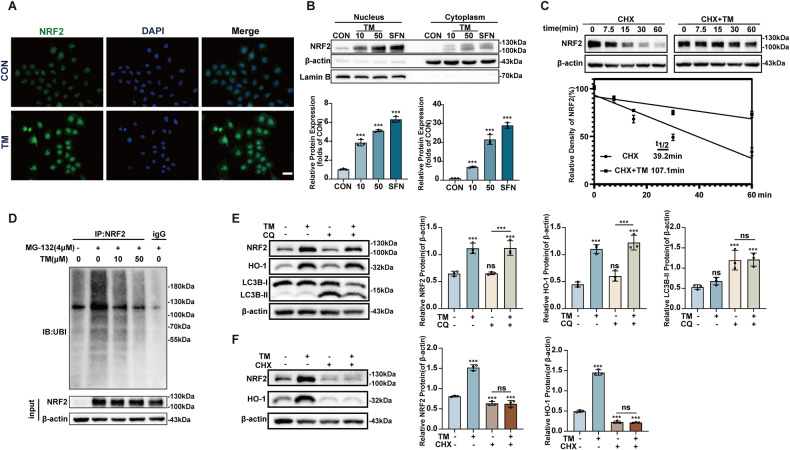
TM Inhibits ubiquitination of NRF2 in HK-2 cells. **A** TM promotes NRF2 nucleus translocation. FITC and DAPI were used to mark NRF2 and the nuclei, respectively. Scale bars: 20 μm. **B** Western blot analysis of nuclear and cytoplasmic cell extracts from HK-2 cells (n = 3). **C** TM elongated the half-life of the Nrf2 protein. Cells were treated with or without TM (50 µM) combine with cycloheximide (CHX, 100 μM) (n = 3). **D** Western blot detected the ubiquitination level of NRF2 in HK-2 cells. **E** The cells were pretreated with chloroquine (CQ) for 1 h, followed with/without TM for 4 h. Protein levels of NRF2, HO-1, LC3B I / II, and β-actin were measured (n = 3). **F** Cells were pretreated with CHX for 1 h, followed with/without TM for 4 h. Protein levels of NRF2, HO-1 and β-actin were measured(n = 3). ns: not significant. * p < 0.05, ** p < 0.01, *** p < 0.001, vs. control group; TM: Ammonium tetrathiomolybdate. Data were presented as the mean ± SD.

The level of NRF2 mRNA was not affected by TM (Fig. 2E), indicating that post-transcriptional modification may confer this situation. Under stress conditions, NRF2 is deubiquitinated, which allows NRF2 to enter the nucleus to promote the downstream products transcription [24]. Immunofluorescence and nuclear and cytoplasmic separation detected a robust enhancement of NRF2 in the nucleus after TM treatment (Fig. 4A, B). Notably, TM treatment significantly prolonged the half-life of NRF2 from 39.2 min to 107.1 min (Fig. 4C). MG132 significantly increased NRF2 ubiquitination, which was hampered by TM treatment (Fig. 4D). Furthermore, we found chloroquine-inhibited autophagy did not affect the effect of TM (Fig. 4E), but cycloheximide completely abolished TM-induced activation of NRF2 (Fig. 4F). These results suggest that TM affects ubiquitination of NRF2 and promotes the accumulation of NRF2 from de novo synthesis.

### TM protects against cisplatin-induced AKI in vivo

**Fig. 5:**
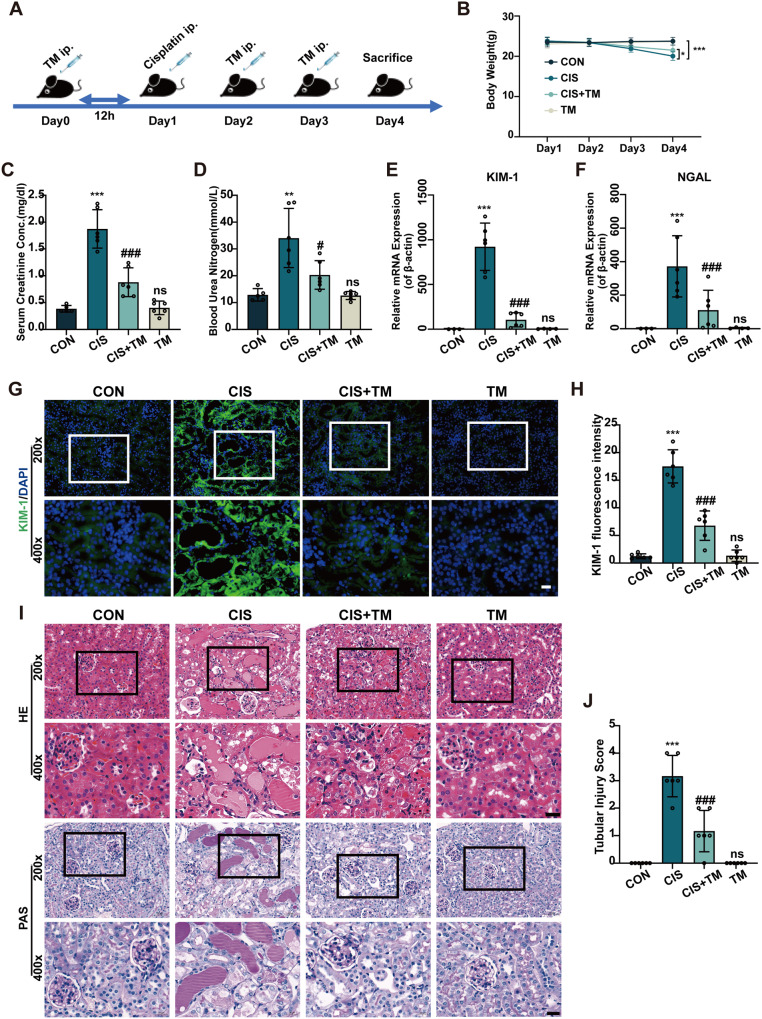
TM treatment ameliorates cisplatin-induced acute kidney injury in mice. **A** Schematic outlining the experimental plan for establishing an animal model: A daily intraperitoneal injection of TM (20 mg/kg) was administered to mice 12 h before the 25 mg/kg dose of cisplatin was administered. **B** Weight changes of mice during treatment (n = 4–6). **C**, **D** Serum creatinine and blood urea nitrogen levels in mice (n = 6). **E**, **F** Expressions of KIM-1 and NGAL in the kidney cortex normalized to β-actin (n = 6). **G** Immunofluorescence for KIM-1(green) of kidney sections and (**H**) quantitative analysis (n = 6). **I** Representative histology of kidney tissues by HE and PAS staining and (**J**) quantitative analysis of tubular injury (n = 6). Scale bars: 20μm. ns: not significant. * p < 0.05, ** p < 0.01, *** p < 0.001, vs. control group; *#p* < 0.05, *##p* < 0.01, *###p* < 0.001, vs. cisplatin group. CON: control; CIS: cisplatin; TM: Ammonium tetrathiomolybdate. Data were presented as mean ± SD.

Next, we investigated the possible effects of TM on cisplatin-AKI in vivo. TM (20 mg/kg) was administered one day prior to cisplatin treatment and then both drugs were administered for three days (Fig. 5A). On the second day, the mice began to lose appetite and weight, which was relieved by TM therapy (Fig. 5B). TM preserved renal function, as shown by lower blood urea nitrogen and serum creatinine (Fig. 5C, D). Furthermore, neutrophil gelatinase-associated lipocalin (NGAL) [25] and kidney injury molecule 1 (KIM-1) [26], which are generally accepted biomarkers of renal injury, increased significantly after exposure to cisplatin and ameliorated by TM treatment (Fig. 5E–H). H&E and PAS staining of renal tissue revealed apparent tubular damage such as tubular dilation, loss of brush border, cytoplasmic vacuoles, and cast formation (Fig. 5I, J). However, these histological abnormalities were attenuated by TM. In addition, long-term observation suggested that TM could significantly reduce the mortality caused by a single high-dose injection of cisplatin in mice (data not shown). Collectively, these findings suggested that TM therapy could reduce cisplatin-induced kidney dysfunction and pathological damage.

### TM treatment alleviates oxidative stress, apoptosis, and inflammatory response in cisplatin-induced AKI animal model

**Fig. 6:**
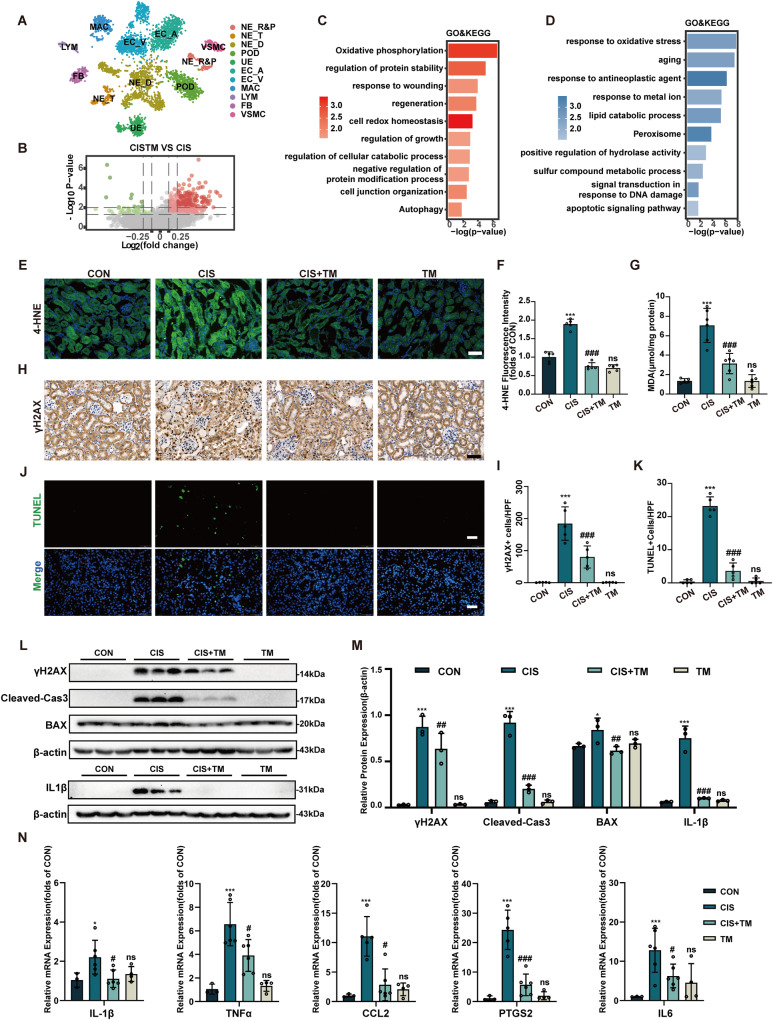
TM relieves oxidative stress, apoptosis, and inflammation in cisplatin-induced AKI. **A** Projection of the tSNE annotated to 11 major cell-type groups: NE: Nephron; R&P: Renal corpuscle and Proximal tubule; T: Thin limb of loop of Henle; D: Distal tubule; POD: podocyte; UE: Ureteric epithelium; EC A&V: Endothelium cells Artery and vein; MAC: Macrophages; LYM: Lymphocytes; FB: Fibroblast; VSMC: Vascular smooth muscle cells. **B** Volcano diagram of different gene expression in NE_R&P cluster between CIS + TM group and CIS group. GO and KEGG enrichment analysis of up-regulated genes (**C**) and down-regulated genes (**D**) between CIS + TM group and CIS group. **E**, **F** Immunofluorescence stain of 4-HNE in mice kidney sections (n = 6). Scale bars: 50 μm. **G** MDA levels of mice kidney tissues. **H**, **I** IHC stain of γH2AX in mice kidney sections (n = 6). Scale bars: 50 μm. **J**, **K** TUNEL staining in the kidney cortex (green: TUNEL positive cells) (n = 5). Scale bars: 50 μm. **L**, **M** Western blot analysis of γH2AX, cleved-caspase3, BAX and IL1β in the kidney cortex (n = 3). **N** Quantitative real-time PCR analysis of IL-1β, TNFα, CCL2, IL6 and PTGS2 in the kidney of mice (n = 6). ns: not significant. * p < 0.05, ** p < 0.01, *** p < 0.01, vs. control group; *#p* < 0.05, *##p* < 0.01, *###p* < 0.01, vs. cisplatin group. CON: control; CIS: cisplatin; TM: Ammonium tetrathiomolybdate. Data were presented as the mean ± SD.

ScRNA-seq analysis was used to assess the effect of TM on cisplatin-mediated transcriptional alterations in the kidney. A total of 3,601 cells passed quality control (Fig. S4B), and unbiased clustering analysis defined 11 cell subtypes (Fig. 6A, S4C). The distribution of different cell types is shown in Fig. S4A. Since cisplatin is absorbed mainly in the proximal tubular epithelium [27], we isolated the renal corpuscle and proximal tubule for future analysis. GO and KEGG terms of DEGs enriched in energy metabolism, oxidative stress, and apoptotic pathway (Fig. S4D, E). Subsequently, we analyzed the up- and down-regulated DEGs between the TM and the cisplatin groups, respectively (Fig. 6B). The up-regulated genes showed elevated protein stability and oxidative homeostasis (Fig. 6C), while the down-regulated genes depicted that TM treatment relieved oxidative stress and the response to antineoplastic agent (Fig. 6D).

Next, we investigated the levels of 4-HNE (Fig. 6E, F) and MDA (Fig. 6G) in kidney tissue, which are lipid peroxidation products that have been linked to pathological alterations in response to oxidative stress [28]. And both the increases in MDA and 4-HNE caused by cisplatin were attenuated upon TM treatment, suggesting that TM can effectively inhibit cisplatin-induced oxidative injury. The DNA damage marker, γH2AX was up-regulated after cisplatin treatment and mitigated by TM administration (Fig. 6H, I). Consistent with γH2AX results, TUNEL staining showed that TM also alleviated DNA damage (Fig. 6J, K). The levels of cleaved-caspase3 and BAX were elevated after cisplatin treatment, but reduced after TM therapy (Fig. 6L, M).

Inflammatory factors play a pivotal role in the progression of AKI. Cisplatin triggers a robust inflammatory response in the kidneys. As shown in Fig. 6L–N, the protein level of IL1β and the mRNA levels of IL-1β, TNF-α, CCL2, IL-6, and PTGS2 were elevated compared to the control group. However, these effects were markedly inhibited by TM. IHC staining of F4/80 (macrophage marker) and ly6g (neutrophil marker) showed that enhanced immune infiltration by cisplatin was significantly rescued after TM treatment (Fig. S3D). These findings show that TM relieves cisplatin-induced oxidative stress, apoptosis, and inflammation in mice.

### TM activates the NRF2 signaling pathway in cisplatin-induced AKI in vivo

**Fig. 7:**
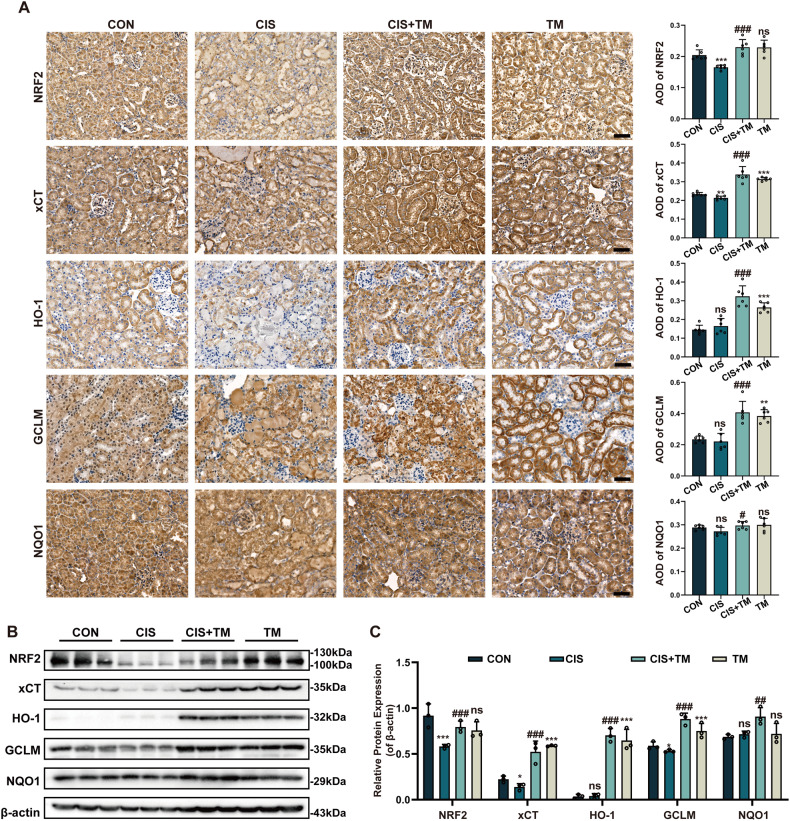
TM activates the NRF2 pathway and boost downstream gene expression in cisplatin-induced AKI. **A** IHC stain of NRF2, xCT, HO-1, GCLM, and NQO1 protein levels in kidney sections from mice. Protein expressions were counted by AOD (n = 6). Scale bars: 50 μm. **B**, **C** Western blot analysis of antioxidant genes downstream of the NRF2 pathway in protein extracted from kidney cortex (n = 3). * p < 0.05, ** p < 0.01, *** p < 0.01, vs. control group; *#p* < 0.05, *##p* < 0.01, *###p* < 0.01, vs. cisplatin group. CON: control; CIS: cisplatin; TM: Ammonium tetrathiomolybdate. Data were presented as mean ± SD.

Finally, we verified whether it could also stimulate NRF2 in renal tissue. Immunohistochemistry (IHC) staining of the sections indicated that NRF2, xCT, HO-1, GCLM, and NQO1 were significantly elevated after TM treatment (Fig. 7A). Consistent with the IHC staining, Western blot examination of kidney protein extracts revealed that TM promoted NRF2 and target proteins (Fig. 7B, C). These findings suggested that TM stimulated the NRF2-regulated antioxidant system in the kidney.

## Discussion

Cisplatin has been used as a standard therapy to treat numerous solid organ malignancies for more than 50 years after the FDA authorized [29]. The most serious adverse effect of cisplatin-based treatment is nephrotoxicity and there are no effective nephroprotectants available in clinical practice [19]. In this study, we demonstrate that TM is a promising nephroprotective drug for the treatment of cisplatin-induced AKI in vivo and in vitro. Furthermore, the protective effect of TM is mediated by NRF2, which suppresses NRF2 ubiquitination and enhances downstream target gene transcription. This pharmacological impact has not been found in previous research, and it would give a therapeutic strategy for the treatment of cisplatin-induced AKI.

Clinically, up to 40% of patients receiving high-dose cisplatin suffer from severe renal dysfunction [30]. Hydration remains the main strategy in clinical settings, but the impact on hemodynamic stability and volume balance restricted the application [31]. Oxidative stress, apoptosis, and inflammation are the main mechanisms of cisplatin nephrotoxicity. And oxidative stress is cited as one of the primary causes of cisplatin-induced AKI. However, very few antioxidants have yet stepped into clinical trials [32]. Even antioxidants that have been shown to be effective in preclinical studies have performed poorly in clinical trials [33]. In our research, we demonstrated that TM can effectively relieve cisplatin-induced oxidative stress in HK-2 cells and mice kidney. TM has been reported to reduce hydroxyl radical production in Wilson disease [34] and superoxide production in ischemia-reperfusion injury [14]. Furthermore, we observed that TM prevents cisplatin-induced apoptosis and inflammation. Although TM has been reported to inhibit the inflammatory response in the LPS model [35] and breast cancer [11], which are considered related to copper chelation. However, the specific molecular mechanism of TM and apoptosis remains unclear. In our cells model, we did not find that excessive copper leads to increased cisplatin toxicity. In addition to the copper component, we also observed that TM was unable to chelate platinum, and platinum chelates have been reported to increase cytotoxicity of cisplatin [18]. Furthermore, recent studies have reported that TM can improve the effectiveness of chemotherapy with cisplatin [36–38]. Renal tubular epithelial cells are the main sites of cisplatin absorption, and robust inflammatory responses and cytokines production originate from renal tubular cells rather than infiltrating immune cells in cisplatin-induced AKI [39, 40]. Thus, the results suggest that TM acts primarily in epithelial cells, at least in our model. Furthermore, we found that TM could activate the NRF2 pathway that is responsible for the protective effect against cisplatin.

Manipulation of NRF2 pathways has been shown to be effective in protecting against various disorders characterized by oxidative damage and inflammation [41], especially during the acute stage of the disease. Systemic NRF2 deletion animals exhibit worsened kidney damage in cisplatin-induced AKI [42]. NRF2 agonists have been extensively investigated, but currently there are few of them that have therapeutic use in clinical applications [5]. Additionally, studies show that TM is a low-toxicity drug. The main side effects occur at high doses (60–80 mg/kg in rats) with long periods of duration (3–6 months) [43]. The dose and duration in our animal trial (20 mg/kg, 3 days) were lower than those of other investigations and no adverse effects were observed. Secondly, NRF2 was activated primarily by TM in the liver and kidney, but had no appreciable impact on the heart, lung, or brain. In paticular, the major site of deposition was the renal tubular epithelial cells [44], which was consistent with our results. It is important to note that many NRF2 activators are currently ineffective in clinical settings due to their off-target effects [41]. While TM possesses effective targeting qualities, it offers a great foundation for clinical use.

**Fig. 8:**
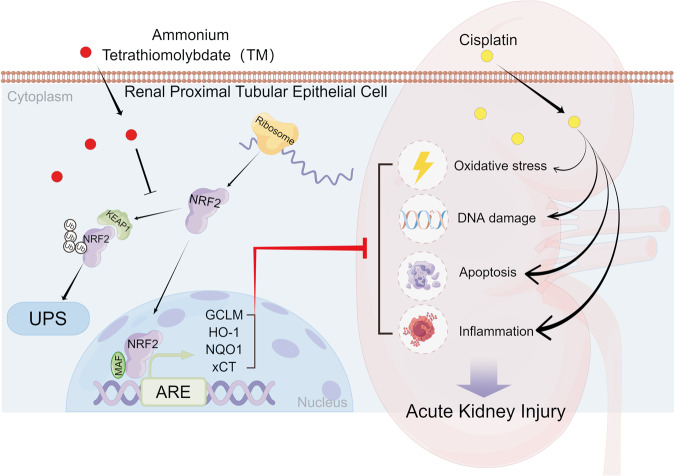
Schematic summary of the mechanism underlying the protective actions of TM in cisplatin-induced AKI. TM inhibits the NRF2 ubiquitination process, resulting in the failure of the newly synthesized NRF2 protein to continue ubiquitination in the UPS system. NRF2 newly synthesized enters the nucleus, binds to the ARE sequence, activates downstream cytoprotective genes, therefore protecting against cisplatin-induced AKI. ARE antioxidant response element. UPS ubiquitin-proteasome system.

As an effective copper chelator, TM has achieved good results in Wilson’s diease [45]. Due to the important role of copper in tumorigenesis and progression [46], TM has been proven to exhibit antineoplastic effects in various malignancies, including breast cancer [36, 47], lung cancer [48], and ovarian cancer [49]. In the present study we reveal a new property of TM which activate the NRF2 singling pathway and coffers the protective effect against cisplatin-induced AKI. Mechanically, NRF2 is mainly degraded by the ubiquitin‒proteasome system(UPS) [7]. We found that TM inhibited the ubiquitination of NRF2. Keap1 is a well-known negative regulator that continuously ubiquitinates NRF2 proteins through a regenerative cycle [50]. Modification of Keap1 domain is a crucial step in the prevention of NRF2 ubiquitination [51]. Our results showed that TM treatment did not significantly change the expression level of KEAP1(data not shown). Another study suggested that the C151S mutation in KEAP1 impaired the NRF2 activation effect of TM. This also implies that KEAP1 is important for TM-induced NRF2 activation. Meanwhile, they found that autophagy modulates TM-induced NRF2 activation in the HUVEC cell line [52]. Our data showed that autophagy inhibition would not mitigate NRF2 activation, but inhibition of protein synthesis totally abrogates the elevation of NRF2 and downstream genes. First, different cell type may explain the different effects of this drug. Second, we speculate that TM prevents the ubiquitination process of NRF2, resulting in non-continuous degradation of newly synthesized NRF2. As a result, de novo NRF2 synthesis accumulates in the cytoplasm and nucleus (Fig. 8). Studies revealed that although NRF2 inducers cause conformational changes in KEAP1, NRF2 remains trapped in blockage complex due to a close conformation. The ubiquitination machinery cannot dissociate, but remains misaligned with NRF2 lysine residues, and the ubiquitination is not feasible [53]. Polysulfides have been observed to alter the cysteine residue of KEAP1, lower ubiquitination levels, and stabilize NRF2 proteins [54–56].

Potential renoprotective strategies for cisplatin nephrotoxicity have been developed in a variety of pharmacological, molecular, and genetic approaches. Clinical translation from bench to bedside remains challenging. In conclusion, the current study provided evidence for additional clinical applications of TM by showing that it activates NRF2 and has a favorable therapeutic impact on cisplatin-induced AKI. However, further research is required to determine how this drug’s pleiotropic properties can be used in particular situations.

## Materials and methods

### Cell culture and reagents

Procell Life Science & Technology supplied human renal proximal tubular epithelial cells (HK-2 cells), which were cultured in Dulbecco’s modified Eagle medium-F-12 (DMEM/F12, Gibco) with 10% fetal bovine serum (FBS), 100 U/ml penicillin and 100 g/ml streptomycin (Gibco) at 37 °C in a cell incubator. HK-2 cells were identified using short tandem repeat (STR) profiling and tested for mycoplasma contamination. Cisplatin (P4394), TM (323446) and copper chloride (229628) were obtained from Sigma-Aldrich. Sulforaphane (TQ0207) and MG-132 (M1902) were sourced from TargetMol. Cycloheximide (M4879) was purchased from Abmole.

### Cell viability assay

Annetta Semisch et al. delineated that copper ions interfere with the reduction of WST-8 and generate a false positive result [57]. Therefore, cell viability was detected using the CellTiter-Lumi^TM^ Plus Luminescent Cell Viability Assay Kit (C0056, Beyotime) according to the manufacturer’s protocol. Briefly, an equal volume of CellTiter-Lumi^TM^ Plus Reagent as culture medium was added to the wells and mixed. The luminous signals were monitored in a microplate reader after another 10 min of incubation at room temperature (EnVision 2105, PerkinElmer).

### ARE-luciferase activity assay

The experimental procedures were carried out using the Dual-Luciferase® Reporter Assay System (E1910, Promega). Cells were transfected with pARE-luc using lipofectamine 3000 transfection reagent (L3000, Thermo Scientific). After being exposed to the indicated drugs, the treatment medium was removed after 12 h. The cells were then collected and placed in the luciferase cell culture lysis solution. Following centrifugation, 20 μL of the supernatant was utilized according to the protocol to measure luciferase activity. A microplate reader was used to measure the luciferase activity. The data was obtained in triplicate and expressed as fold induction over control.

### RNA Isolation and Quantitative Real-Time PCR

The Total RNA Kit (R6834, Omega) was used to extract total RNAs from HK-2 cells following the manufacturer’s protocol. RNAiso Plus (9108Q, Takara) was used to extract kidney mRNA. The HiScript III RT SuperMix for the qPCR kit (R323, Vazyme) was used to perform reverse transcription according to the manufacturer’s descriptions. Quantitative real-time PCR was performed with ChamQ Universal SYBR qPCR Master Mix (Q711-02, Vazyme). The gene primers used are listed in supplementary tables 1-2. The ΔΔCt method was used to obtain the relative level of target genes normalized against β-actin.

### Western blot

Kidney tissue and HK-2 cells were harvested and lysed with RIPA lysis buffer (CW2333S, CWbio) containing a protease inhibitor cocktail (CW2200, CWbio). A BCA protein assay kit (BL521A, Biosharp) was used to determine the concentration. An equivalent quantity of protein was separated on a 7.5 ~ 12% SDS-PAGE gel (1610173, Bio-Rad) and transferred to PVDF membranes (ISEQ00010, Millipore). The membranes were blocked and probed with various primary antibodies overnight at 4 °C. After incubation, HRP-conjugated secondary antibody was used at room temperature for 1 h. Primary antibodies are shown in supplementary table 3. Proteins were visualized using enhanced chemiluminescence (WBULS0100, Millipore) and quantified using ImageJ software.

### Immunofluorescence

After being exposed to TM for the appropriate periods, HK-2 cells were fixed, permeabilized, and incubated with primary antibodies at 4 °C overnight. Following a PBS wash, cells were incubated with a secondary goat anti-rabbit IgG antibody (SA00003-1, Proteintech) for 1 h at 37 °C. To see the nuclei, cells were then labeled with DAPI and examined using a laser scanning confocal microscope (FV1000, Olympus) with a maximum excitation wave length of 570 nm and 340 nm.

### Transfection of small interference RNA (siRNA)

Gene Pharma provided predesigned siRNA for the silencing of human NRF2 and control scrambled siRNA. Oligos were transfected into cells using lipofectamine 3000 transfection reagent according to the manufacturer’s protocol. The RNAi sequences used in the present study are shown as follows: siNrf2-1. F: GGUUGAGACUACCAUGGUUTT; siNrf2-1. R: AACCAUGGUAGUCUCAACCTT; siNrf2-2. F: CCAGAACACUCAGUGGAAUTT; siNrf2-2. R: AUUCCACUGAGUGUUCUGGTT; siNrf2-3. F: GCCUGUAAGUCCUGGUCAUTT; siNrf2-3. R: AUGACCAGGACUUACAGGCTT.

### Nuclear and cytoplasmic extraction

HK-2 cells were seeded in 10 cm dishes with a density of 1× 10^7^ cells. Nuclear and cytoplasmic pieces were separated according to the NE-PER™ Nuclear and Cytoplasmic Extraction Reagent’s instructions (78833, Thermo Fisher). Then an immunoblot analysis assay was used to measure the different protein levels in the nuclear and cytoplasmic fractions.

### Mitochondrial membrane potential and mtROS measurement

The mitochondrial membrane potential (MMP) of HK-2 cells was detected by a JC-1 fluorescent probe (HY-15534, MCE). After incubation with JC-1 solution for 15 min at 37 °C, cells were washed three times. The cells were examined using a laser scanning confocal microscope. Mitochondrial ROS (mtROS) were measured using the MitoSOX™ Red mitochondrial superoxide indicator (M36007, Invitrogen) according to the manufacturer’s instructions.

### Flow cytometric detection of apoptosis and ROS level

A six-well tissue culture plate containing HK-2 cells was treated with TM and cisplatin at the indicated doses for the indicated times. After treatment, cells were washed, resuspended in binding buffer, and stained with Annexin V/PI (A213, Vazyme) for apoptosis detection and CM-H2DCFDA (C6827, Invitrogen) for ROS level detection according to the manufacturer’s instructions. The stained cells were analyzed by flow cytometry (CytoFLEX, Beckman Coulter) and the data were determined using FlowJo VX software.

### H_2_S detection assay

The free sulfide (H_2_S gas) liberated from TM was measured following the procedure described previously [17]: As shown in Fig. S1C, a solution of TM was placed in a sealed 1.5 ml Eppendorf vial. WSP-5(GC45162, GlpBio), a H_2_S-specific fluorescent probe, was also placed in the 0.2 ml vial to trap the evaporated H_2_S from the TM solution. The trapping solution was collected and the fluorescence intensity (excitation at 502 nm, emission at 525 nm) was evaluated after 8 h of incubation at 37 C. To test the protective effect of hydrogen sulfide released from TM, as shown in Fig. S1D, HK-2 cells seeded in a 2 cm dish with a density of 1×10^5^ cells, then placed this dish in a 6 cm dish containing 3 ml of TM solution at the indicated concentration. After 24 h of incubation, the growth state of the cells was imaged under a standard light microscope.

### Immunoprecipitation (IP)

Immunoprecipitation was carried out using the Pierce Co-Immunoprecipitation Kit (26149, Thermo Fisher). Briefly, after treatment with the indicated drug, immunoprecipitation was performed using an anti-NRF2 antibody overnight at 4 °C. The next day, after elution, anti-ubiquitin primary antibodies were used for western blot analysis.

### RNA Sequencing

RNA was isolated from HK-2 cells using TRIzol reagent (15596026, Invitrogen), following the manufacturer’s instructions, and sent to Guangzhou IGE biotechnology LTD for library preparation and sequencing. The FPKM value was adopted to calculate gene expression. For differential expression analysis, P ≤ 0.01, and |Log2 (fold change) |≥1.5 were set as inclusion criteria. KEGG analysis was performed with a public online database. GSEA (Gene Set Enrichment Analysis) was performed with the WIKIPATHWAY dataset. Accession numbers are listed in the supplementary table S4.

### Animal experiments

All mice were purchased from the Guangdong Provincial Medical Laboratory Animal Center, and animal experiments were approved by the Ethics Committee of the Fifth Affiliated Hospital at Sun Yat-sen University, Guangdong, China. C57BL/6 J mice (male, 6–8 weeks old, 20–25 g) were acclimated for three days at a temperature of 22 °C and a relative humidity of 60% while fed regular laboratory mouse meal and water. The sample sizes for which (typically 6 animals) were based on prior experience. And mice were randomly assigned to different groups by animal care technicians not involved in the experimental studies. The investigators were not blinded to allocation during experiments and outcome assessment. During the experiment, the mice had unrestricted access to food and water. Body weight was recorded each day. The animals were randomly assigned to one of the four treatment groups (6 animals in each group): (A) control group; (B) cisplatin group; (C) cisplatin + TM group; and (D) TM group. Briefly, Cisplatin (25 mg/kg i.p. injection) was given as a single injection and TM (20 mg/kg i.p. injection) [14] was given 12 h before cisplatin administration and once daily thereafter. Mice were anesthetized by inhalation of isoflurane and sacrificed on the third day after cisplatin injection. Renal cortical tissues and blood samples were collected and stored appropriately for further analysis. All the experiments were replicated at least three times. Data were only excluded when technical failures occurred.

### Measurement of creatinine and urea nitrogen detection in blood serum

The whole blood was quiescence at room temperature for 40 min and centrifuged at 4 °C,4000 rpm, for 20 min to acquire the serum sample. Creatinine in serum was determined using Creatinine Assay Kit (DICT-500, Bioassay) and urea nitrogen was determined using the Urea Assay Kit (C013, Nanjing Jiancheng bioengineering institute), following the manufacturer’s instructions.

### Histopathological scoring

After being embedded in paraffin, sectioned and stained with HE, renal tissue was observed under an optical microscope. Histopathological scoring was performed using a blind method and the score was assessed by grading tubular necrosis, loss of brush border, cast formation, and tubular dilatation. A score of 0 to 4 was given for tissue pathological damage: 0, no abnormalities; 1 + , <25%; 2 + , 25–50%; 3 + , 50–75%; 4 + , > 75%. At least five renal regions on each slide were randomly selected.

### Measurement of MDA Levels

The renal tissue collected from the mice was homogenized by tissue homogenizer. After centrifugation at 10,000× g for 30 min, the supernatant was analyzed using an MDA assay kit (M496, Dojindo) following the manufacturer’s protocol. MDA levels were normalized by protein concentration.

### Immunohistochemistry

Immunohistochemistry (IHC) was performed following the instructions of the SABC anti-rabbit-POD kit (SA1028, Boster Bioengineering). Briefly, paraffin sections were dewaxed with gradient ethanol, steam heated for antigen retrieval in EDTA-based buffer, blocked with 3% H_2_O_2_ followed by 5% BSA. The specimens were then incubated with primary antibody at 4 °C overnight, rewarmed to 37 °C for 1 h and washed with PBS. Subsequently, tissue slices were exposed to drop-wise additions of biotinylated IgG as secondary antibodies for 30 min at 37 °C. After incubation with SABC substance for 30 min at 37 °C, 3,3′-Diaminobenzidine (DAB) was used for color reaction. Slides were counterstained with hematoxylin, mounted, and imaged under a standard optical microscope. Densitometry analysis was performed by Image J.

### Terminal deoxynucleotidyl transferase dUTP nick-end labeling (TUNEL) assay

The TUNEL assay was performed using the TUNEL BrightGreen Apoptosis Detection Kit (A112, Vazyme) according to the manufacturer’s protocol. Briefly, kidney sections were deparaffinized and rehydrated. Subsequently, the sections were incubated with the TUNEL reagent mixture for 60 min and washed with PBS. Nuclei were stained with DAPI and images were observed using fluorescence microscopy. TUNEL-positive cells were expressed as a percentage of total cells.

### Single-cell RNA-seq

All single cell dissociation and sequencing was performed by Biomarker Technologies Corporation according to the manufacturer’s protocol. Briefly, kidneys were collected and digested into a single-cell suspension. Cell debris was removed using the Miltenyi debris removal solution. Cells were diluted to a concentration of 10^6^cells/ml. Single cells, reagents and a single gel bead containing cell-barcoded were encapsulated into nanoliter-sized Gel Beading Emulsion by using BMKMANU-DG1000 platform. And the cDNA libraries were constructed using the Chromium Single Cell 3′ Library & Gel Bead Kit v3. The Agilent bioanalyzer high sensitivity DNA test was used for qualitative analysis. The final libraries from NC group (CON), cisplatin group (CIS), and cisplatin+TM group (CISTM) were sequenced on an Illumina NovaSeq 6000 sequencer. After quality control by using Seurat package in R, STAR software was used to process single-cell RNA sequencing output and align the read to the mouse reference transcriptome (mm10-3.0.0). The Seurat integration strategy was performed based on the work of Andrew Ransick et al. [58] to identify common cell types and enable comparative analyzes. The Accession numbers are listed in supplementary table 4.

### Quality control, cell identification, and clustering analysis

The gene cell matrix of each sample was utilized to make a Seurat object using the Seurat package in R. The following threshold criteria were used to further select cells: the total number of expressed genes, 300–9000; total UMI count, <20,000; and proportion of mitochondrial genes expressed, <30%. According to the ‘package handbook’, batch correction was carried out utilizing the Seurat package’s IntegrateData function. The percentages of nFeature (number of Gene), nUMI (number of UMI) and percent.mt (mitochondrial gene) of each sample cell were counted (Fig. S4B).

### Differentially expressed gene calculation and pathway analysis

The Seurat function FindAllMarkers was used to identify differentially expressed genes (DEGs) based on the normalized UMI count. GO analysis was performed using the “Web Gestalt [website http://www.webgestalt.org]”, Based on log2(FC) and P-values of DEGs, the pathway analysis was carried out using the Ingenuity Pathway Analysis (IPA).

### Statistics and reproducibility

All statistical analyzes were performed with the GraphPad Prism program (Version 8.0, USA) and quantitative data were reported as mean + standard deviation (SD). All assays were conducted at least in triplicate. The data meet the normal distribution. And the variances between the groups being statistically compared are similar. Statistical differences between two groups were determined using the two-tailed unpaired Student’s t-tests. Tukey’s post-tests and one-way ANOVA were used to determine statistical differences between various groups. Bonferroni corrections for multiple comparisons were performed. P-value less than 0.05 was considered statistically significant. N values are reported for each experiment in the figure legend.

## Supplementary information

Supplementary figures

Supplementary tables

Original Data File

## Data Availability

The data of this study are available in the article and supplementary materials.
