# Supplementary material for: Ammonium tetrathiomolybdate relieves oxidative stress in cisplatin-induced acute kidney injury via NRF2 signaling pathway
Source: Cell Death Discov. 2023 Jul 25;9:259. doi: 10.1038/s41420-023-01564-1 (PMC10368633; doi:10.1038/s41420-023-01564-1)
Supplement: Supplementary file 1 — Supplementary figures [file 41420_2023_1564_MOESM1_ESM.docx]

**Ammonium** **Tetrathiomolybdate Relieves Oxidative Stress in Cisplatin-induced Acute Kidney Injury via NRF2 Signaling Pathway**

**Supplementary Figures**

Hao Qi^1,2^, Haoyu Shi ^1,2^, Minbo Yan^1,2^, Liangyu Zhao^1,2^, Yinghao Yin ^1,2^, Xiaolin Tan^3^, Huiyue Qi^1,2^, Hu Li^1,2^, Kangqiang Weng^1,2^, Yuxin Tang ^1,2^, Yingbo Dai ^1,2#^

^1^Department of Urology, The Fifth Affiliated Hospital of Sun Yat-Sen University, Zhuhai, China.

^2^Guangdong Provincial Key Laboratory of Biomedical Imaging, The Fifth Affiliated Hospital of Sun Yat-Sen University, Zhuhai, China.

^3^Department of Clinical Nutrition, The Fifth Affiliated Hospital of Sun Yat-Sen University, Zhuhai, China.

# Correspondence:

Yingbo Dai (daiyingbo@126.com),

1. Department of Urology, The Fifth Affiliated Hospital of Sun Yat-sen University, No. 52 Mei Hua Dong Road, Zhuhai, China, 519000；

2. Guangdong Provincial Key Laboratory of Biomedical Imaging, The Fifth Affiliated Hospital, Sun Yat-sen University, Zhuhai, China.


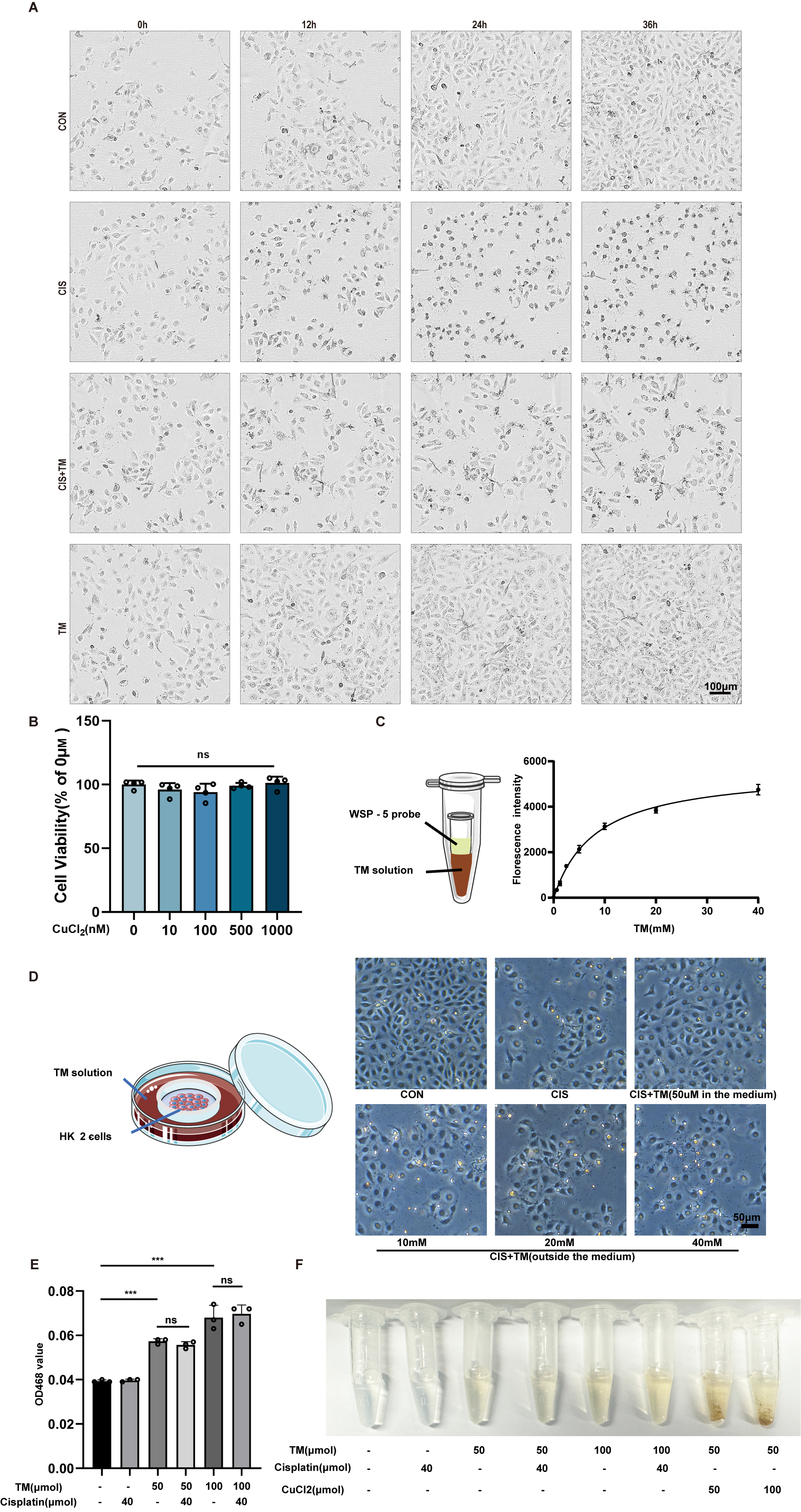


**Supplementary Fig. 1.**

(**A**) Morphological change of HK-2 cells after treatment. Scale bars: 100 μm. (**B**) Copper chloride showed no cytotoxicity in the concentration range 0-1000 nM (n=4). (**C**) WSP-5 assay detected that TM releases hydrogen sulfide in a concentration-dependent manner (n=3). (**D**) Strategy to explore the direct protective effect of H_2_S: After 24h incubation, H_2_S exhibited no direct protective effect on cisplatin induced injury in HK-2 cells. (**E**) Mixing cisplatin with TM does not increase absorbance of OD_468_. (**F**) TM mixed with cisplatin does not produce precipitation, copper chloride served as a positive control


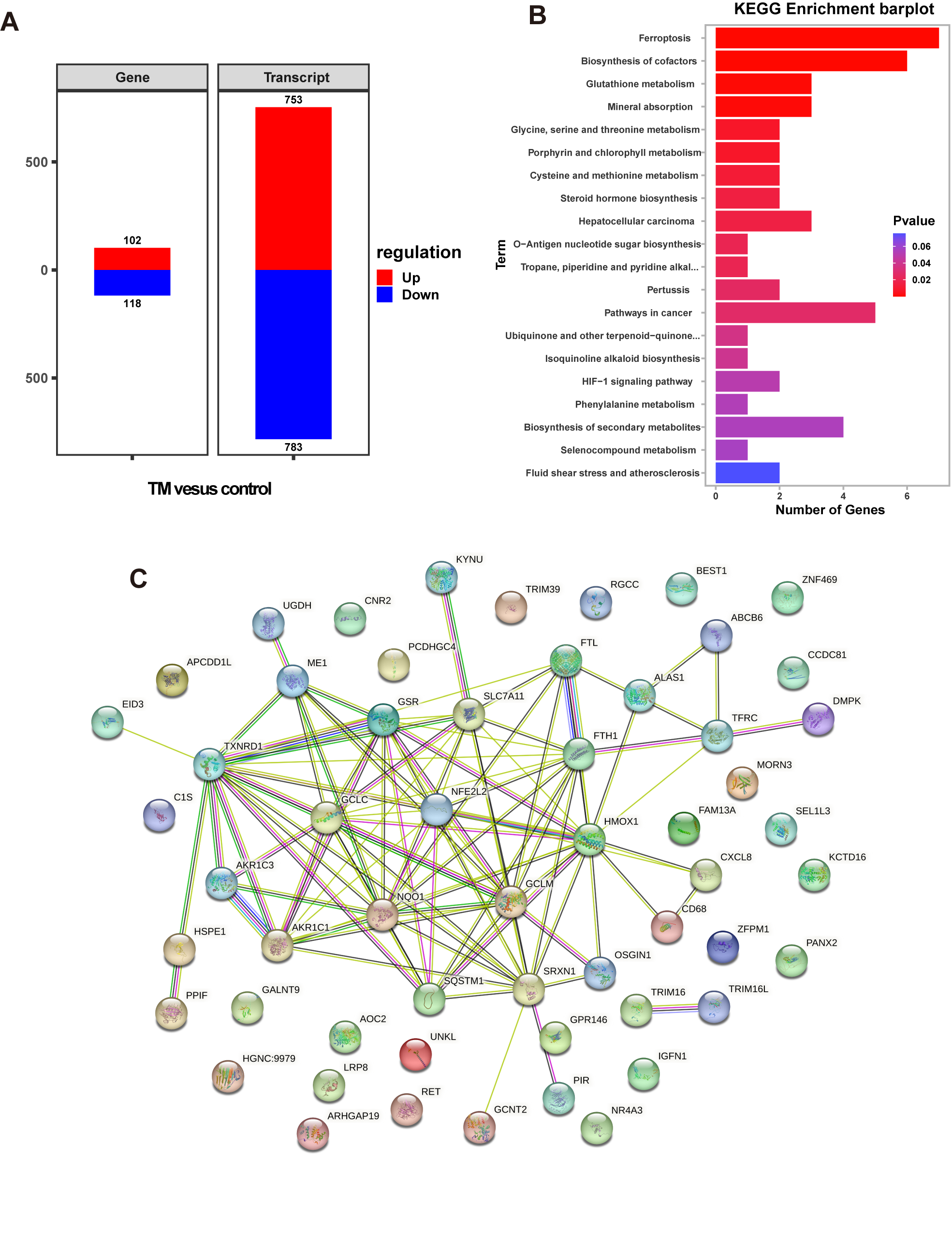


**Supplementary Fig. 2.**

(**A**) Number of differential express genes by transcriptome sequencing between TM group and control group. (**B**)KEGG enrichment of up-regulated genes in TM treatment group in HK-2 cells. (**C**) PPI network of up-regulated genes through STRING


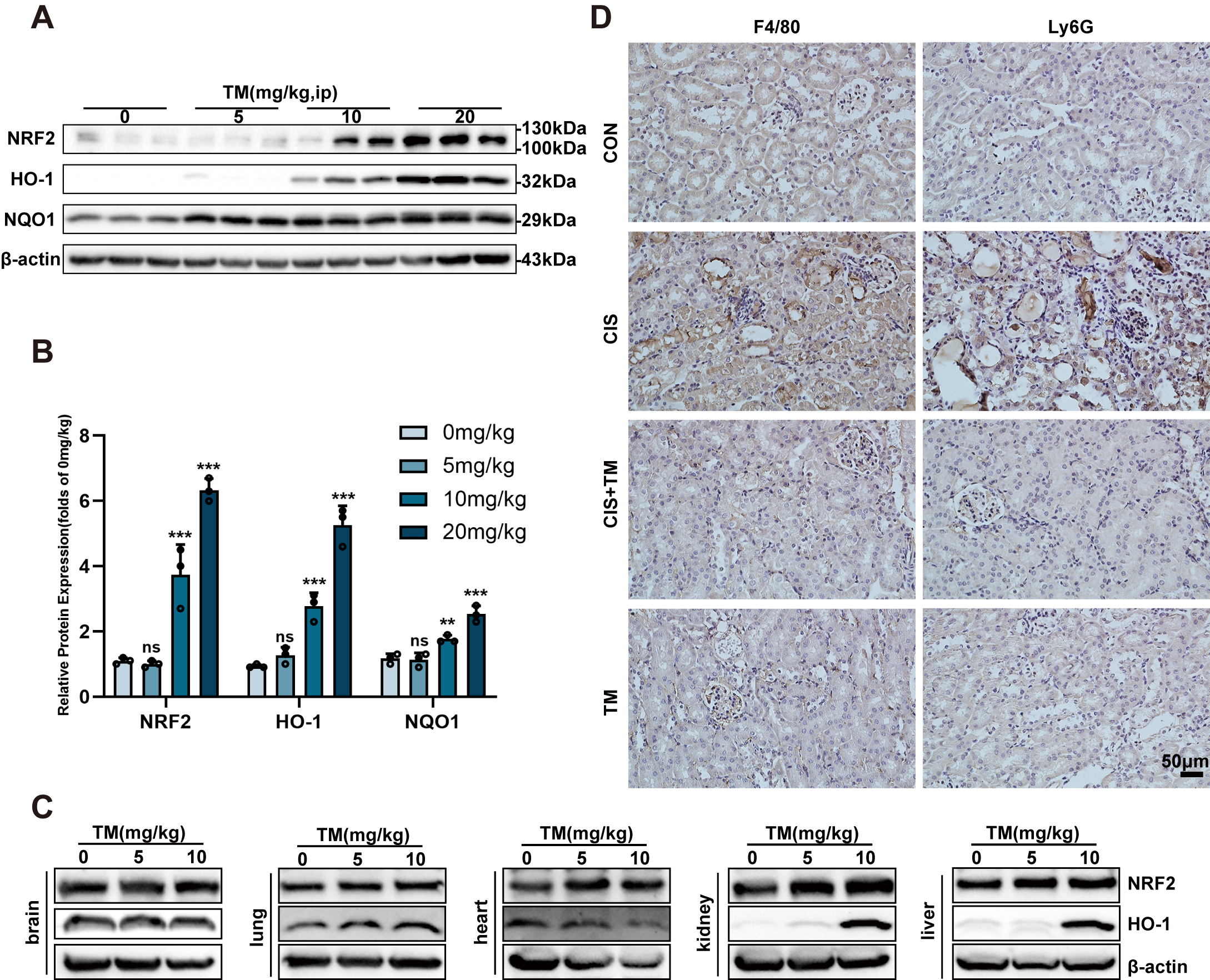


**Supplementary Fig. 3.**

(**A-B**) The effect of TM in mouse kidney. Mice were intraperitoneally injected with the indicated dose and sacrificed 24 hours later to extract kidney cortex protein (n=3). (**C**) The effect of TM in different organs of mice (n=3). (**D**) Immunohistochemical stain of F4/80 and Ly6G in kidney section. Scale bars: 50 μm. Data are presented as means ± SD, statistical analysis was performed by one-way ANOVA followed by Tukey’s test, ** p < 0.01, *** p < 0.01 vs. 0mg/kg group.


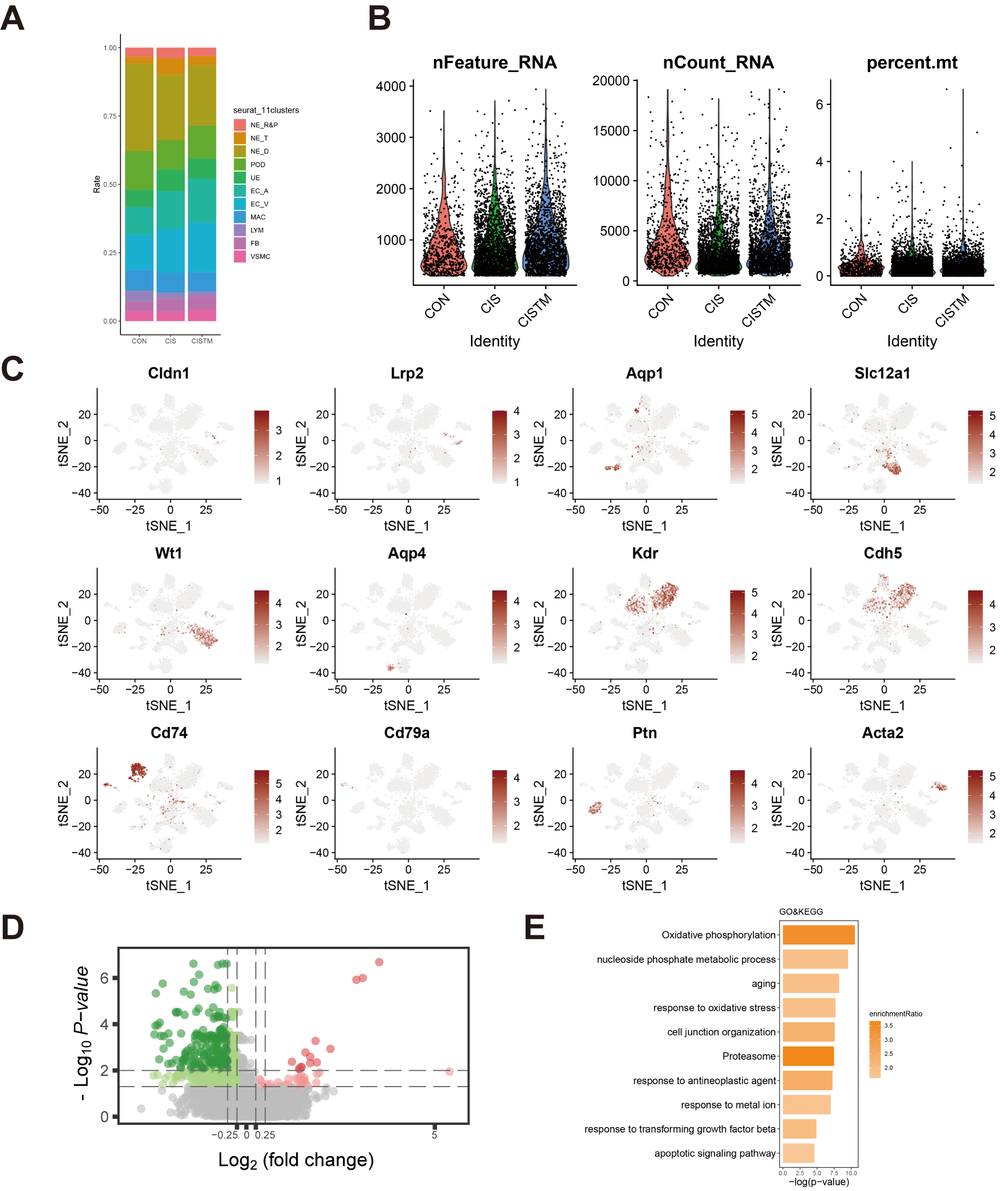


**Supplementary Fig. 4.**

**(A)** Stacked barplots illustrating the composition of clusters of different cell type in different samples. **(B)** Violin plots showing gene counts(left), UMI counts(middle) and percent mitochondrial genes(right) of different samples. **(C)** Markers used to identify common cell types. (**D**)Volcano plot of DEGs between control group and cisplatin group. (**E**) GO&KEGG enrichment analysis of DEGs between control group and cisplatin group.
