# Supplementary material for: Ammonium tetrathiomolybdate relieves oxidative stress in cisplatin-induced acute kidney injury via NRF2 signaling pathway
Source: Cell Death Discov. 2023 Jul 25;9:259. doi: 10.1038/s41420-023-01564-1 (PMC10368633; doi:10.1038/s41420-023-01564-1)
Supplement: Supplementary file 2 — Supplementary tables [file 41420_2023_1564_MOESM2_ESM.docx]

**Ammonium** **Tetrathiomolybdate Relieves Oxidative Stress in Cisplatin-induced Acute Kidney Injury via NRF2 Signaling Pathway**

**Supplementary Tables**

Hao Qi^1,2^, Haoyu Shi ^1,2^, Minbo Yan^1,2^, Liangyu Zhao^1,2^, Yinghao Yin ^1,2^, Xiaolin Tan^3^, Huiyue Qi^1,2^, Hu Li^1,2^, Kangqiang Weng^1,2^, Yuxin Tang ^1,2^, Yingbo Dai ^1,2#^

^1^Department of Urology, The Fifth Affiliated Hospital of Sun Yat-Sen University, Zhuhai, China.

^2^Guangdong Provincial Key Laboratory of Biomedical Imaging, The Fifth Affiliated Hospital of Sun Yat-Sen University, Zhuhai, China.

^3^Department of Clinical Nutrition, The Fifth Affiliated Hospital of Sun Yat-Sen University, Zhuhai, China.

# Correspondence:

Yingbo Dai (daiyingbo@126.com),

1. Department of Urology, The Fifth Affiliated Hospital of Sun Yat-sen University, No. 52 Mei Hua Dong Road, Zhuhai, China, 519000；

2. Guangdong Provincial Key Laboratory of Biomedical Imaging, The Fifth Affiliated Hospital, Sun Yat-sen University, Zhuhai, China.

**Table S1** Primers for real-time quantitative PCR analysis in HK-2 cells.

| Genes | Primer | Sequence |
| --- | --- | --- |
| NRF2 | Forward | 5′- TCAGCGACGGAAAGAGTATGA -3′ |
|  | Reverse | 5′- CCACTGGTTTCTGACTGGATGT-3′ |
| FTH1 | Forward | 5′-TGAAAGCTCTCCACCTCCAGGGACA-3′ |
|  | Reverse | 5′-GAGGCCCAAGGCCACAGGTATTTTG-3′ |
| SCL7A11 | Forward | 5'-TAAGTGCGATTGTACCCGGAC-3' |
|  | Reverse | 5'-TTTGTAGCCATAGTCAGCATTGT-3' |
| NQO1 | Forward | 5'- GAAGAGCACTGATCGTACTGGC-3' |
|  | Reverse | 5'- GGATACTGAAAGTTCGCAGGG-3' |
| GCLM | Forward | 5'- CATTTACAGCCTTACTGGGAGG-3' |
|  | Reverse | 5'- ATGCAGTCAAATCTGGTGGCA-3' |
| HMOX1 | Forward | 5'- AAGACTGCGTTCCTGCTCAAC-3' |
|  | Reverse | 5'- AAAGCCCTACAGCAACTGTCG-3' |
| IL1β | Forward | 5'-GTGGAAACCCACAACGAAAT-3' |
|  | Reverse | 5'-CACGTGCTGCTCCACTTTTA-3' |
| IL6 | Forward | 5'- ACTCACCTCTTCAGAACGAATTG -3' |
|  | Reverse Reverse s | 5'- CCATCTTTGGAAGGTTCAGGTTG -3' |
| TNFα | Forward | 5'- CCTCTCTCTAATCAGCCCTCTG -3' |
|  | Reverse | 5'- GAGGACCTGGGAGTAGATGAG -3' |
| IL8 | Forward | 5'- TTTTGCCAAGGAGTGCTAAAGA -3' |
|  | Reverse | 5'- AACCCTCTGCACCCAGTTTTC -3' |
| β-actin | Forward | 5'- CATGTACGTTGCTATCCAGGC -3' |
|  | Reverse | 5'- CTCCTTAATGTCACGCACGAT -3' |

**Table S2** Primers for real-time quantitative PCR analysis in mice.

| Genes | Primer | Sequence |
| --- | --- | --- |
| KIM-1 | Forward | 5′- GGAAGTAAAGGGGGTAGTGGG-3′ |
|  | Reverse | 5′- AAGCAGAAGATGGGCATTGC-3′ |
| NGAL | Forward | 5′- GCCCAGGACTCAACTCAGAA-3′ |
|  | Reverse | 5′- GACCAGGATGGAGGTGACAT-3′ |
| IL1β | Forward | 5′- GCAACTGTTCCTGAACTCAACT -3′ |
|  | Reverse | 5′- ATCTTTTGGGGTCCGTCAACT -3′ |
| TNFα | Forward | 5′- CCCTCACACTCAGATCATCTTCT -3′ |
|  | Reverse | 5′- GCTACGACGTGGGCTACAG -3′ |
| CCL2 | Forward | 5′- TTAAAAACCTGGATCGGAACCAA-3′ |
|  | Reverse | 5′- GCATTAGCTTCAGATTTACGGGT-3′ |
| IL6 | Forward | 5′- CCAAGAGGTGAGTGCTTCCC -3′ |
|  | Reverse | 5′- CTGTTGTTCAGACTCTCTCCCT -3′ |
| PTGS2 | Forward | 5'- TGAGCAACTATTCCAAACCAGC -3' |
|  | Reverse | 5'-TACACCGACCCACCGAAGACACAG-3' |
| β-actin | Forward | 5'- GCACGTAGTCTTCGATCACTATC -3' |
|  | Reverse | 5'- GCCGGACTCATCGTACTCC -3' |

**Table S3** Antibodies used in study

| Antibodies | Source | Identifier |
| --- | --- | --- |
| β-actin | Abclonal | AC026 |
| γH2aX | Abclonal | AP0687 |
| HO-1 | Abclonal | A19062 |
| NRF2 | Proteintech | 16396-1-AP |
| Lamin-B1 | Proteintech | 12987-1-AP |
| GCLM | Proteintech | 14241-1-AP |
| NF-κB p65 | Proteintech | 10745-1-AP |
| NQO1 | Cell Signaling Technology | 62262 |
| xCT/SLC7A11 | Cell Signaling Technology | 12691 |
| LC3A/B | Cell Signaling Technology | 4108 |
| Phospho-NF-κB | Cell Signaling Technology | 3033T |
| Cleaved Caspase-3 | Cell Signaling Technology | 9664S |
| F4/80 | Cell Signaling Technology | 30325t |
| IL-1β | Santa Cruz Biotechnology | sc-12742 |
| Ubiquitin(P4D1) | Santa Cruz Biotechnology | sc-8017 |
| Ly6G | Abcam | ab238132 |
| 4-HNE | Abcam | ab46545 |
| KIM-1 | Abcam | ab228973 |
| HRP Goat Anti-Rabbit IgG (H+L) | Abclonal | AS014 |
| HRP Goat Anti-Mouse IgG (H+L) | Abclonal | AS003 |

**Table S4** Raw sequencing data in The Sequence Read Archive.

| Sample | Accession Number |
| --- | --- |
| HK-2 cells transcriptome sequencing data | |
| NC1 | [SRR24793423](https://dataview.ncbi.nlm.nih.gov/object/SRR24793423) |
| NC2 | SRR24793422 |
| NC3 | SRR24793421 |
| TM1 | SRR24793420 |
| TM2 | SRR24793419 |
| TM3 | SRR24793418 |
| Mice kidney single-cell sequencing data | |
| Control | SRR24820317 |
| Cisplatin | SRR24820316 |
| Cisplatin+TM | SRR24820315 |
