# Supplementary material for: Ammonium tetrathiomolybdate relieves oxidative stress in cisplatin-induced acute kidney injury via NRF2 signaling pathway
Source: Cell Death Discov. 2023 Jul 25;9:259. doi: 10.1038/s41420-023-01564-1 (PMC10368633; doi:10.1038/s41420-023-01564-1)

**Fig. 1.J-K**

Cleaved-CAS3


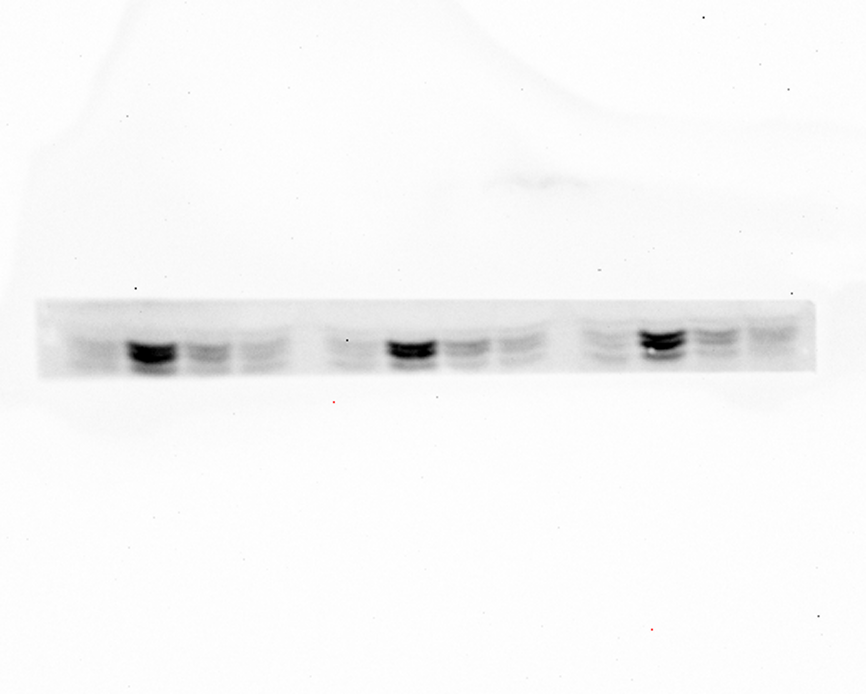


H2AX


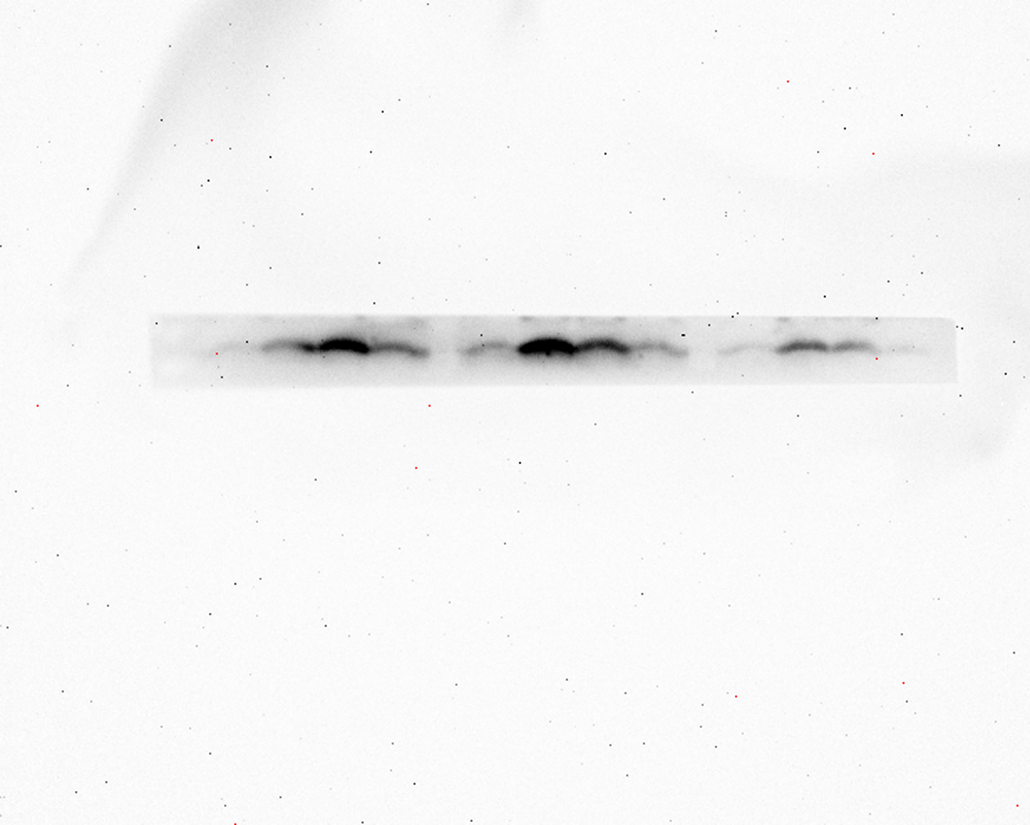


β-actin


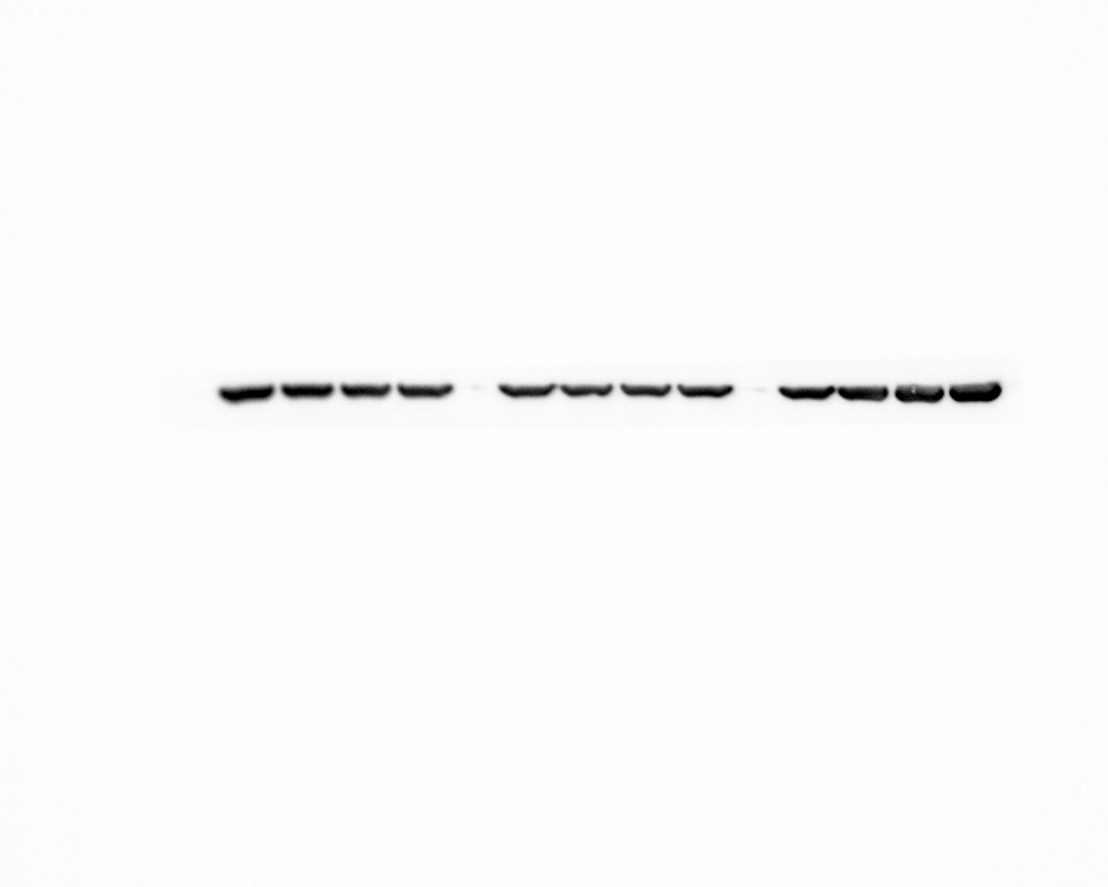


NFKB


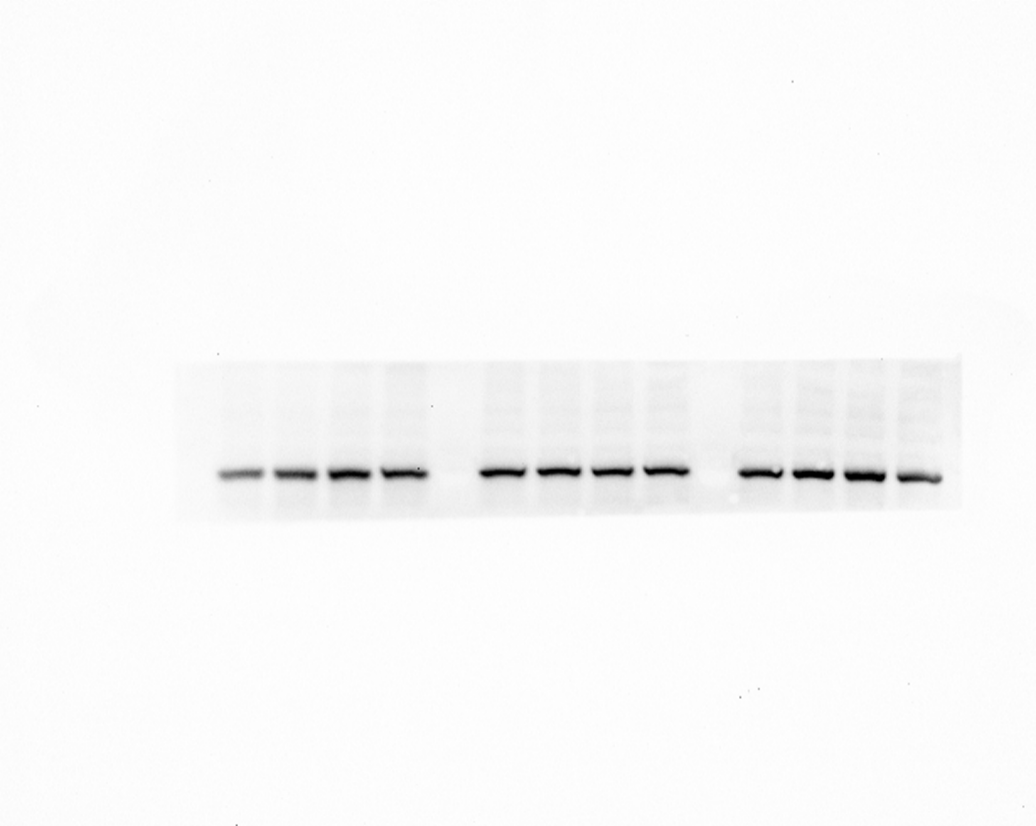


p-NFKB


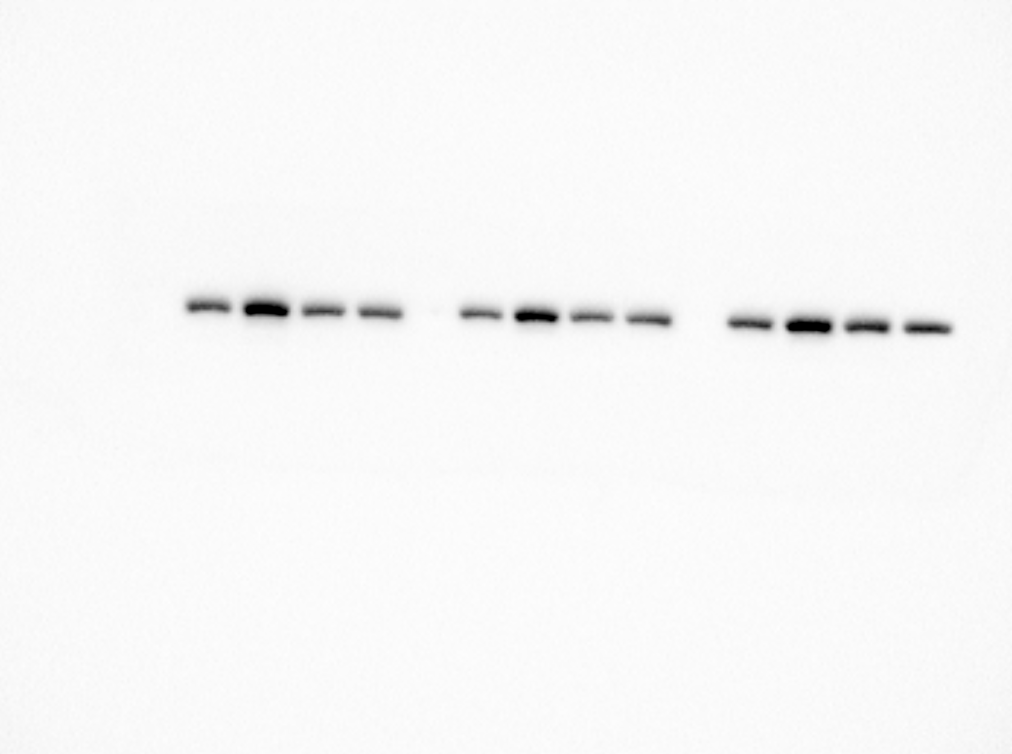


β-actin


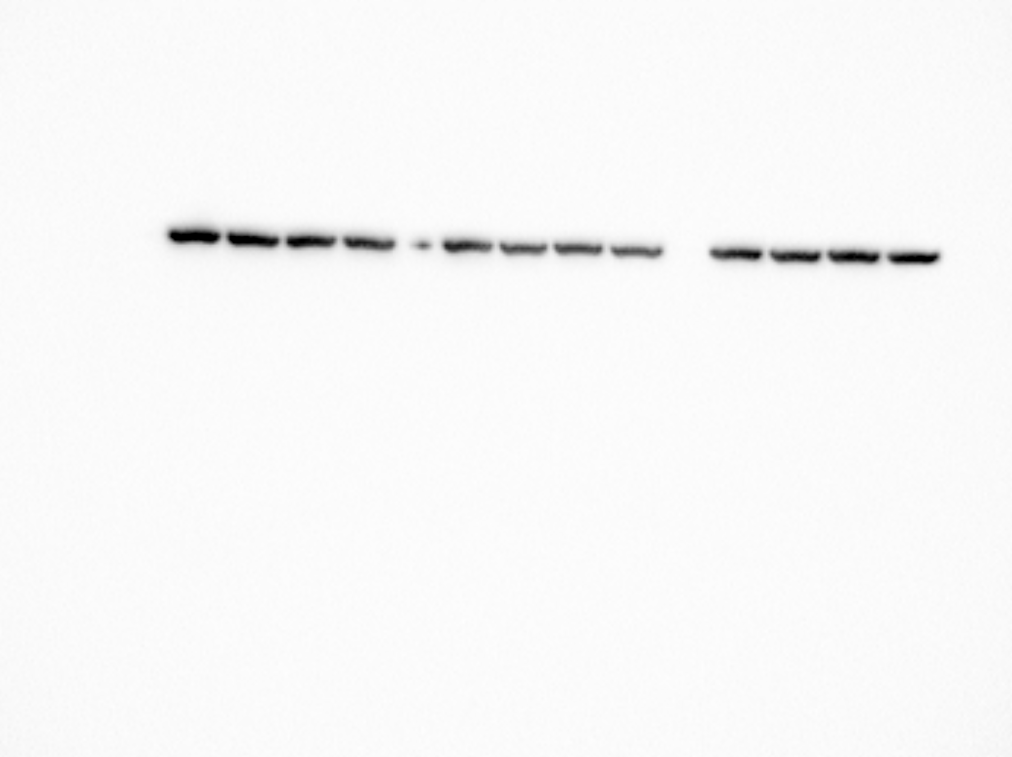


**Fig. 2. G**

NRF2


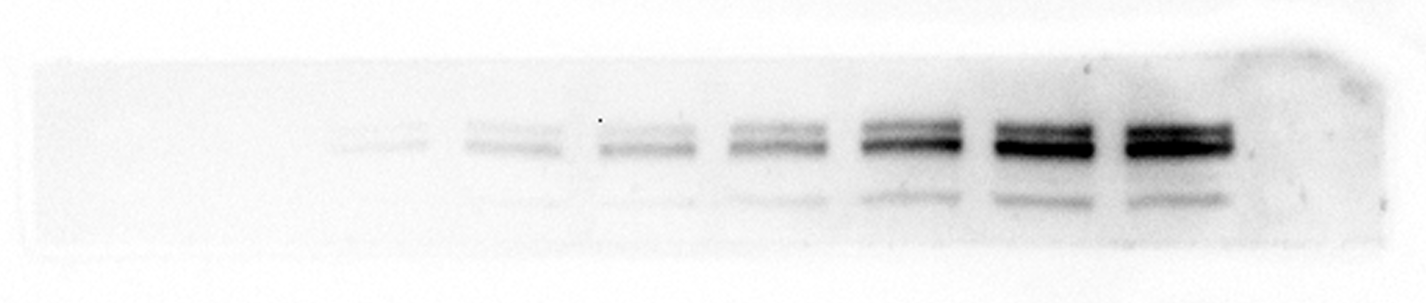


NQO1


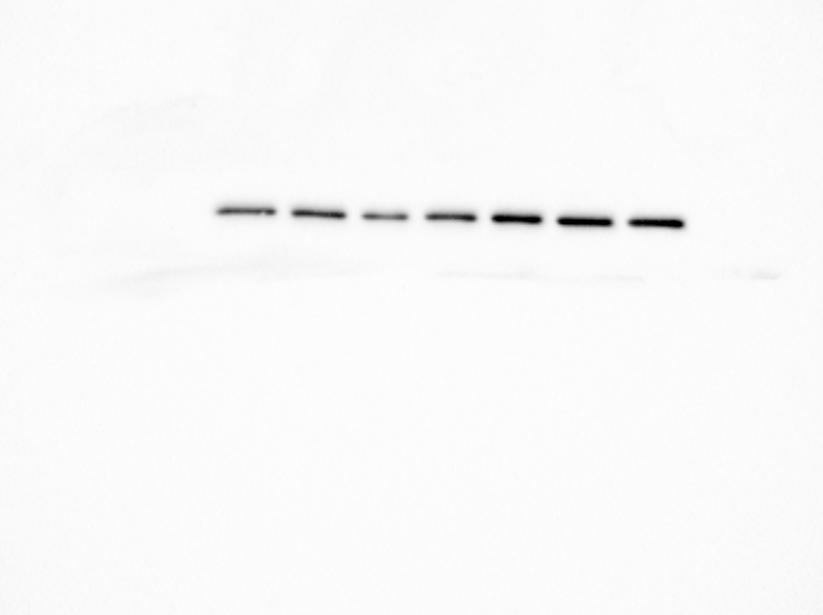


xCT


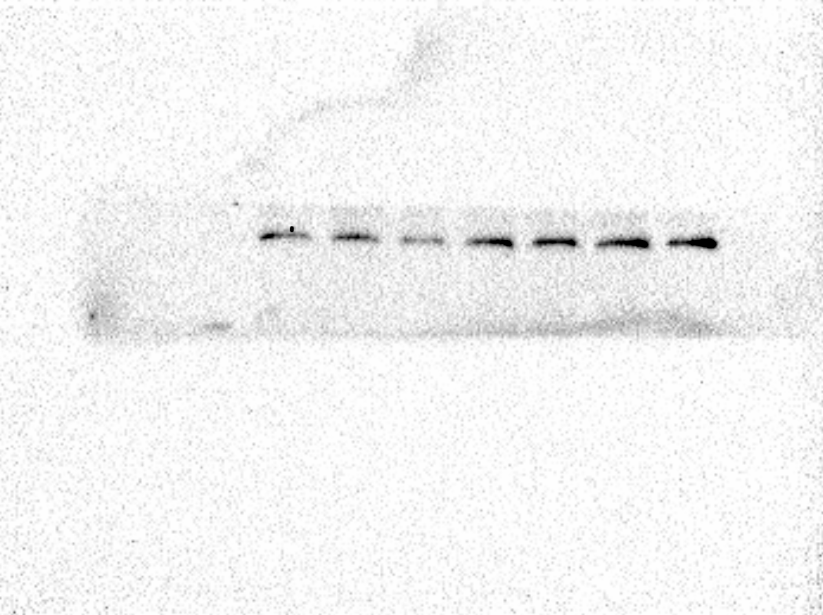


HO-1


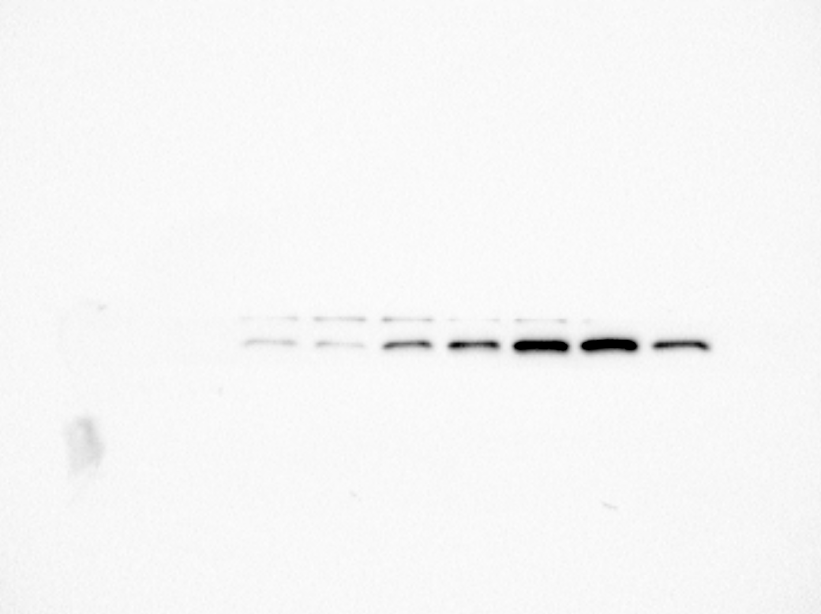


GCLM


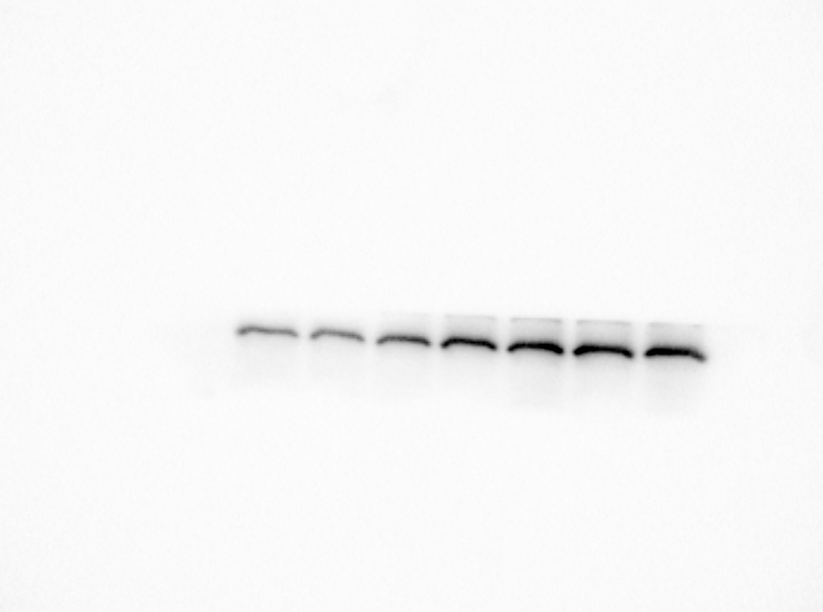


β-actin


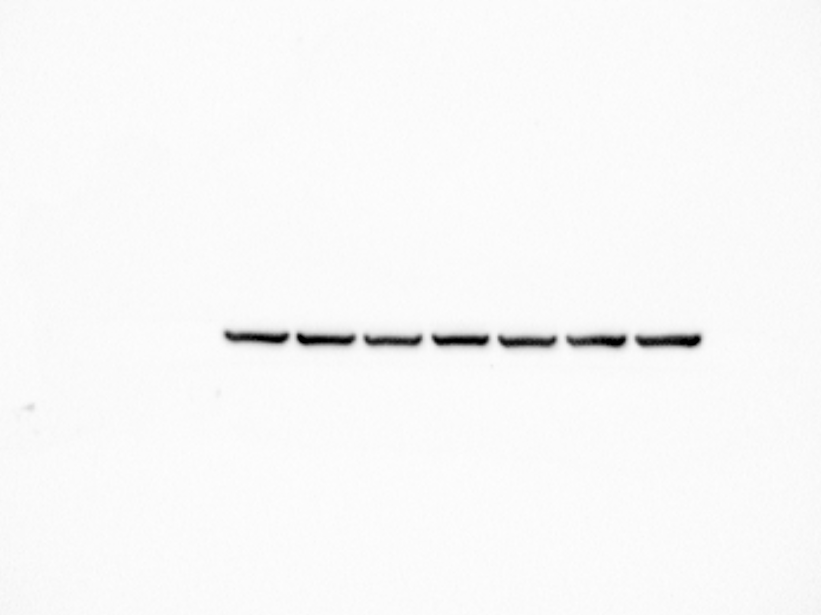


**Fig. 3.A**

NRF2


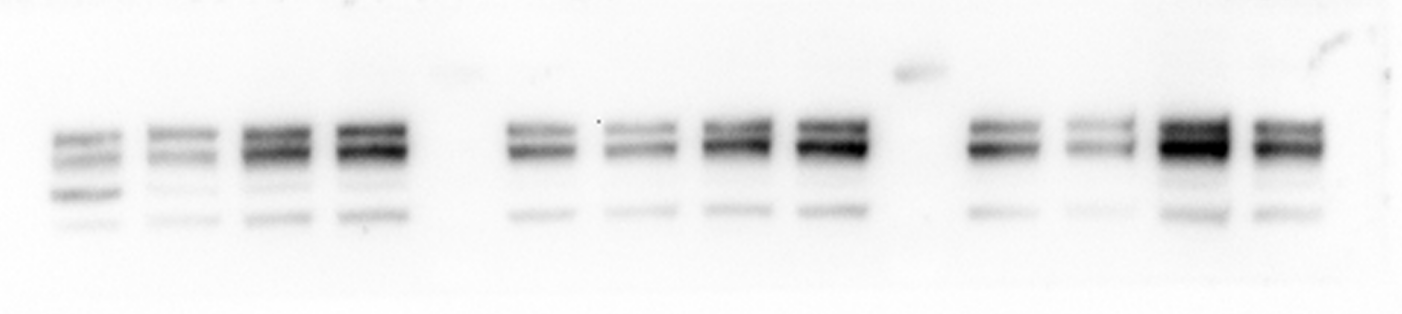


GCLM


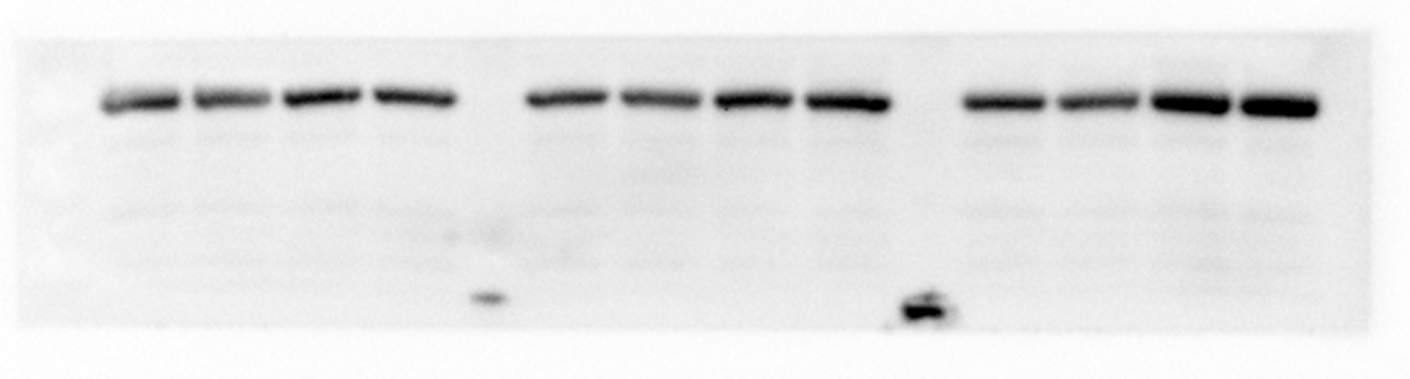


HO-1


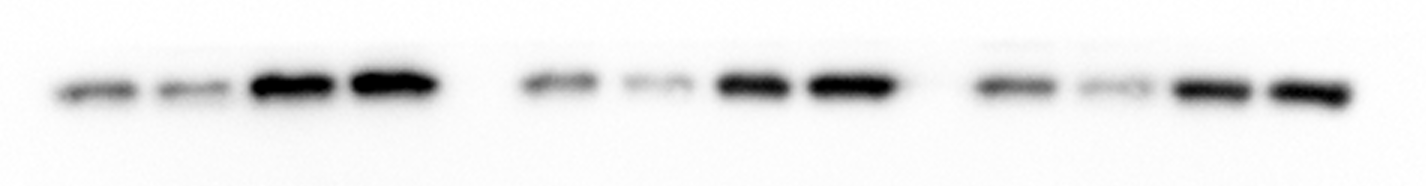


NQO1


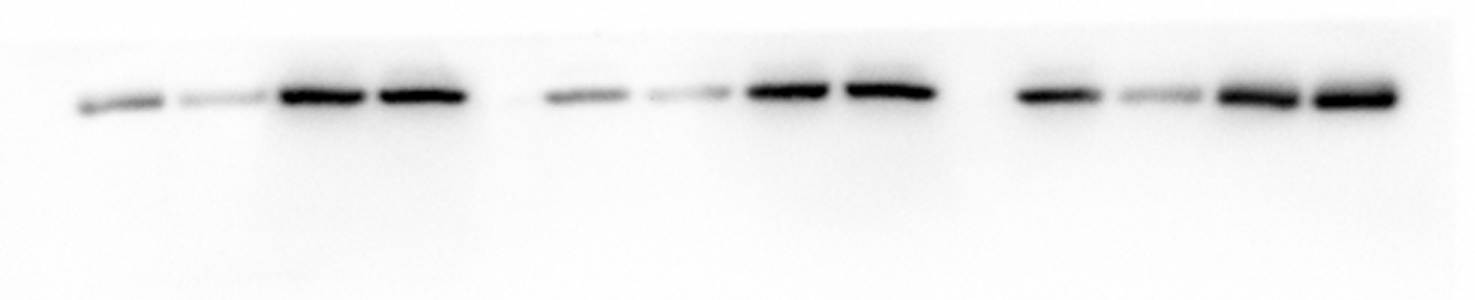


xCT


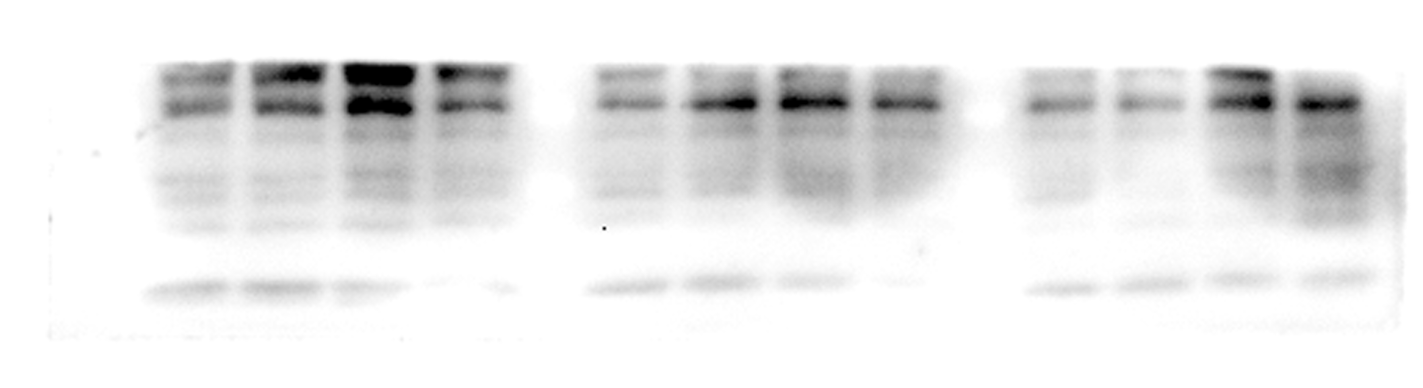


βACTIN


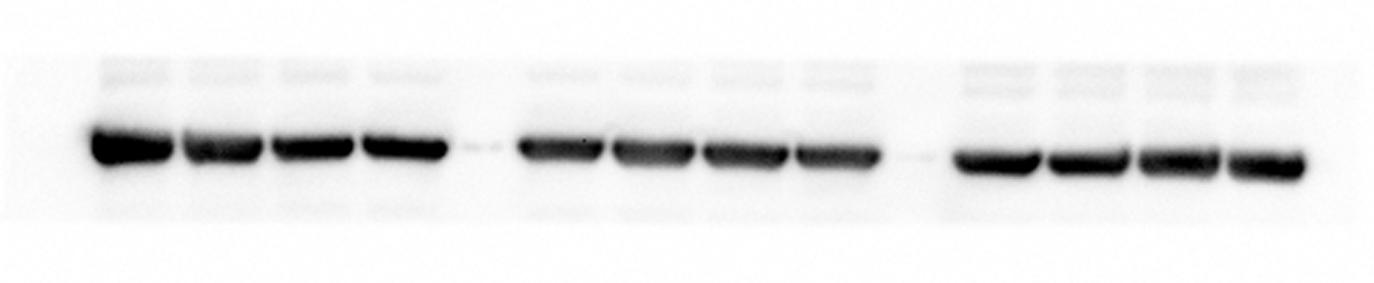


**Fig. 3 C**

NRF2


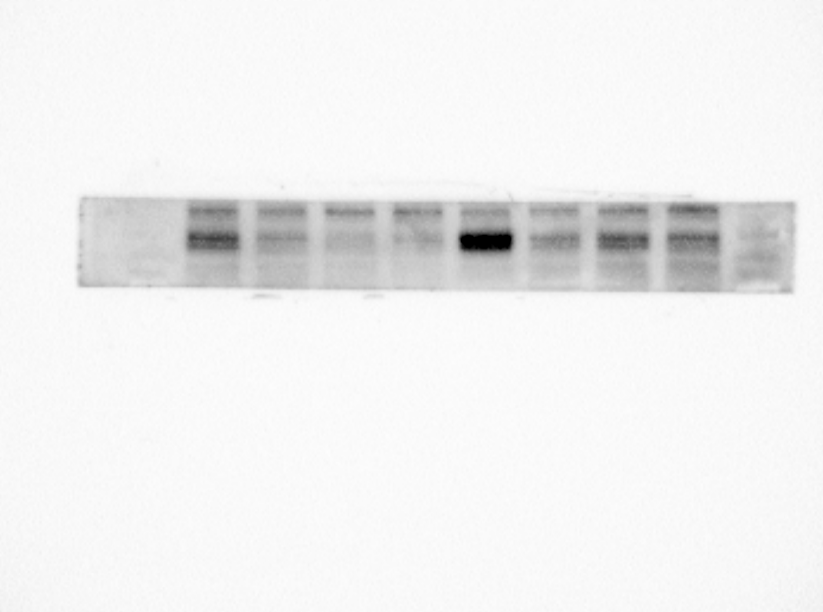


GCLM


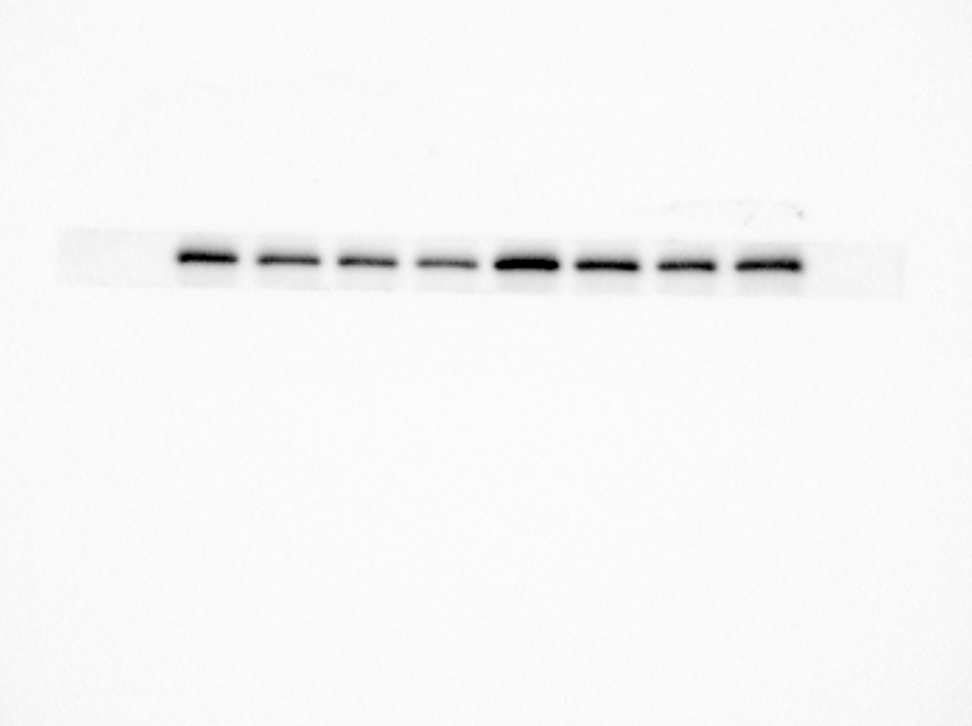


HO1


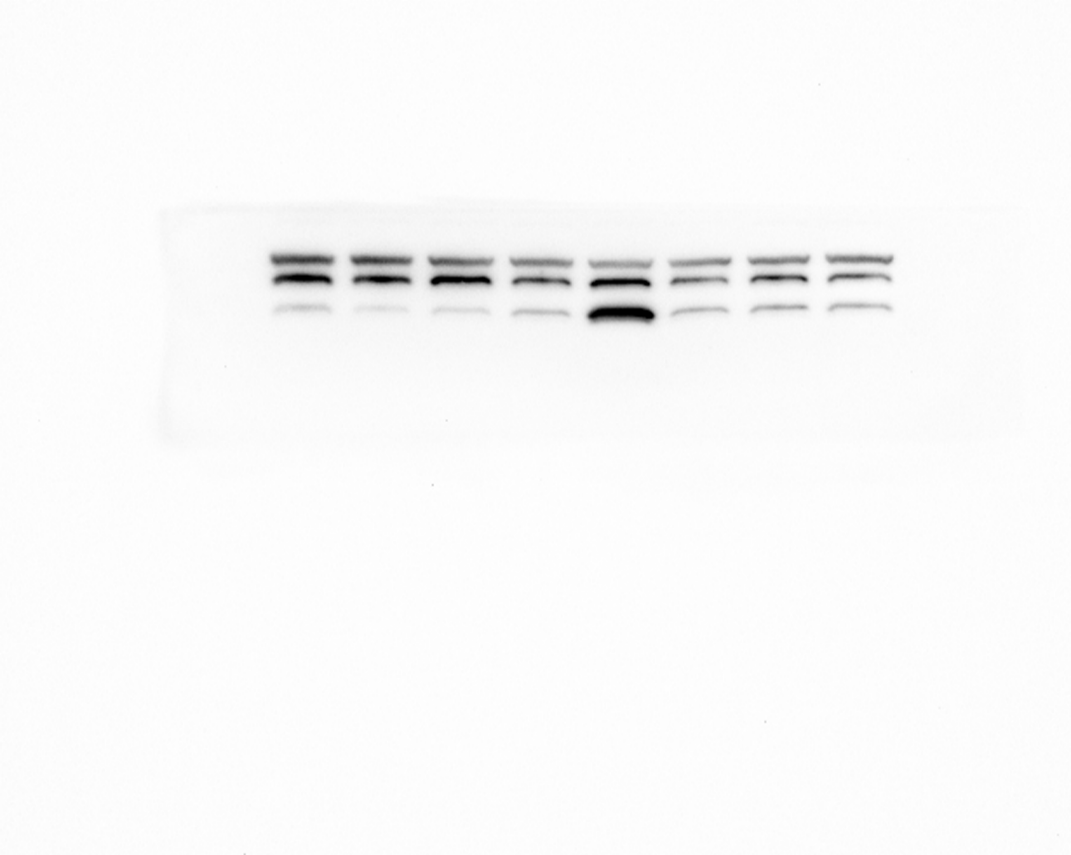


NQO1


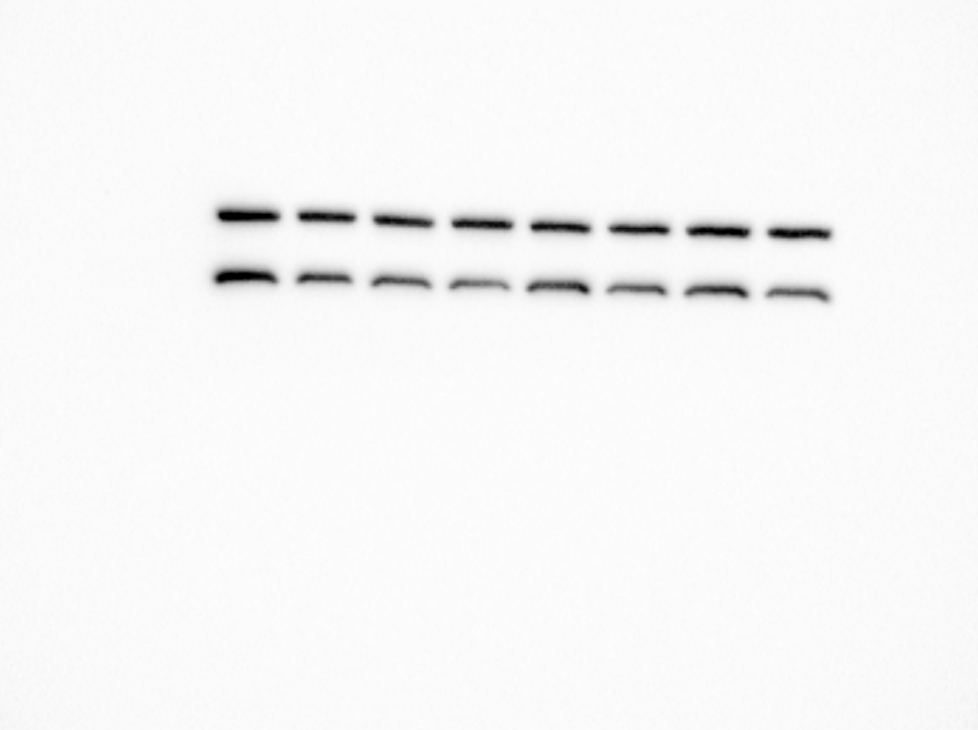


Xct


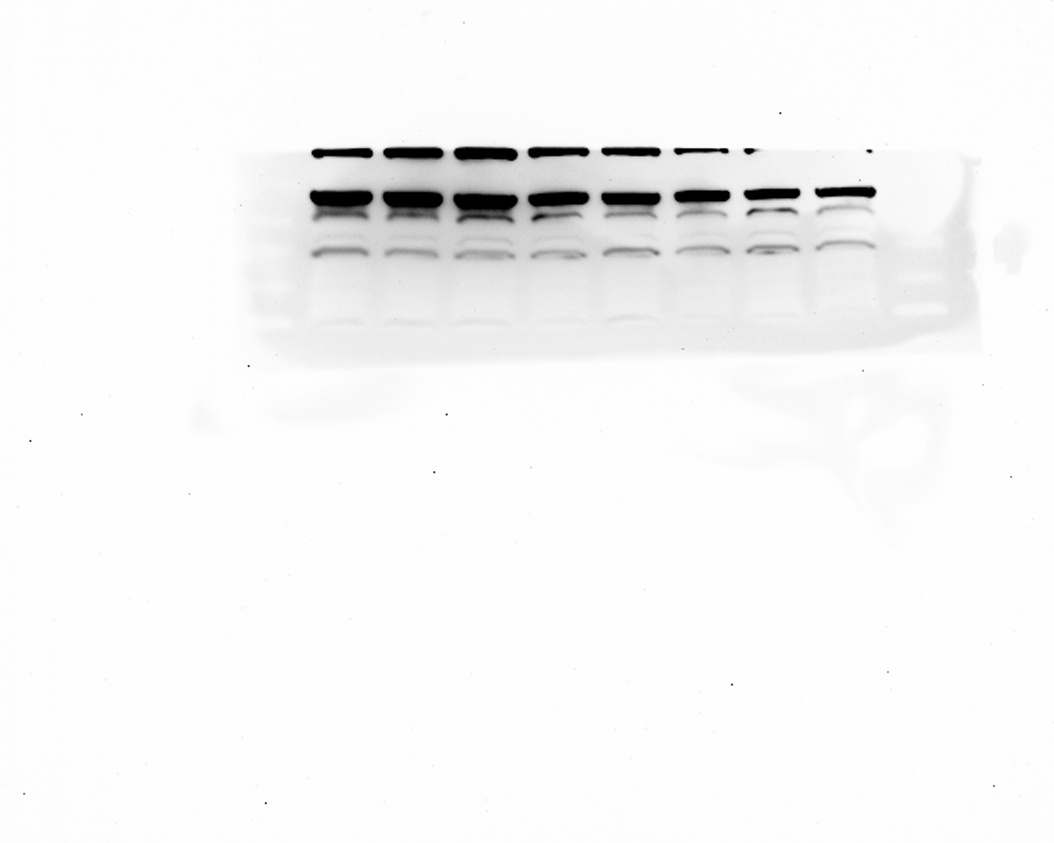


β-ACTIN


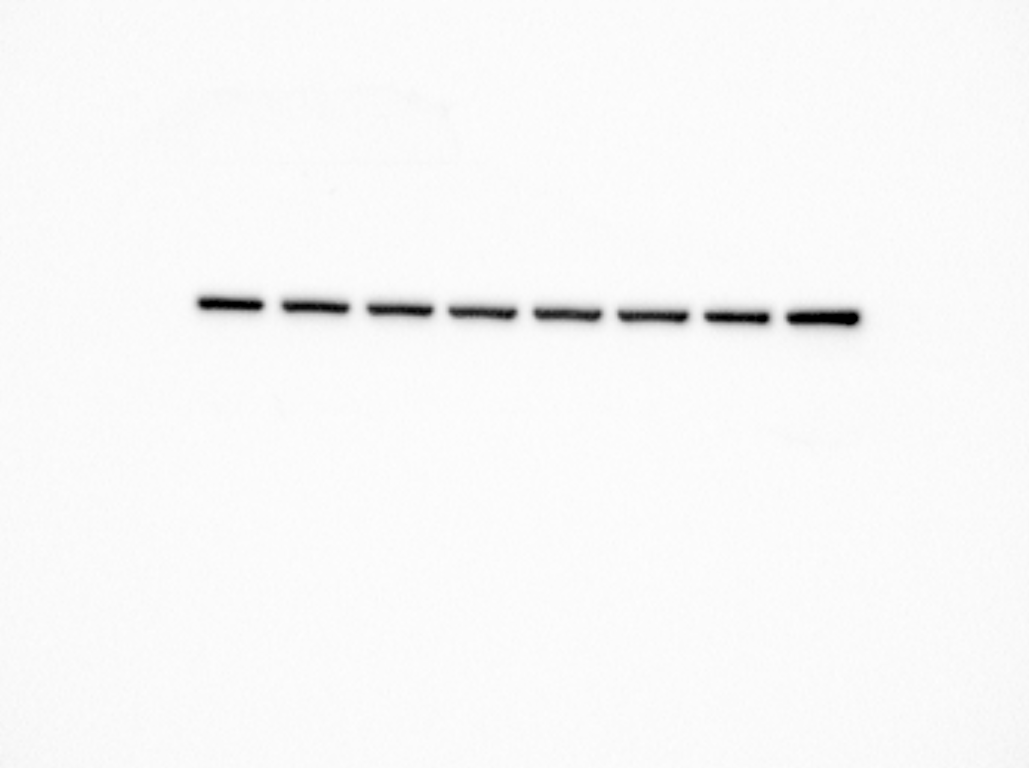


**Fig. 4.C**

NRF2


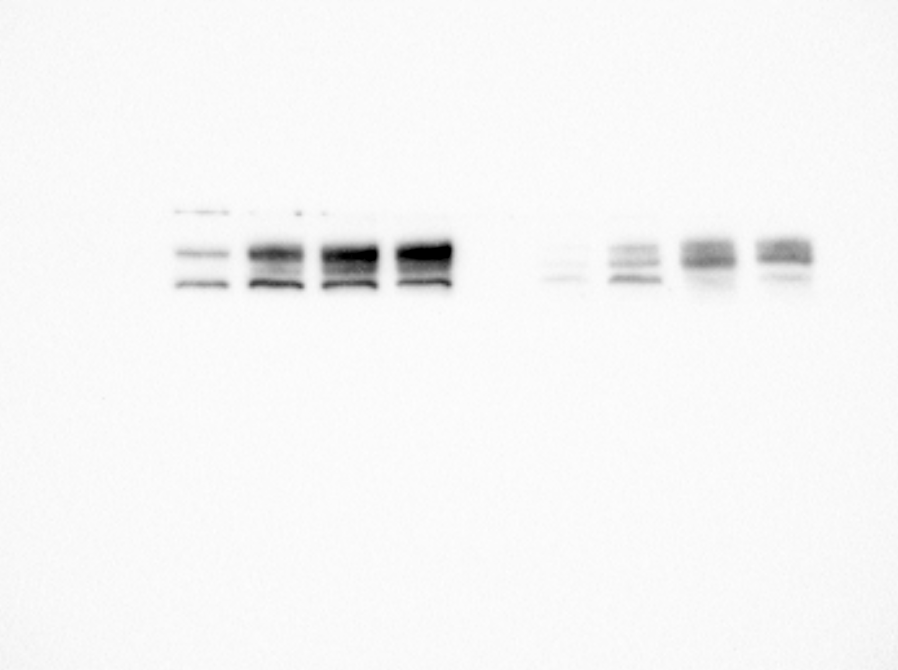


β-actin


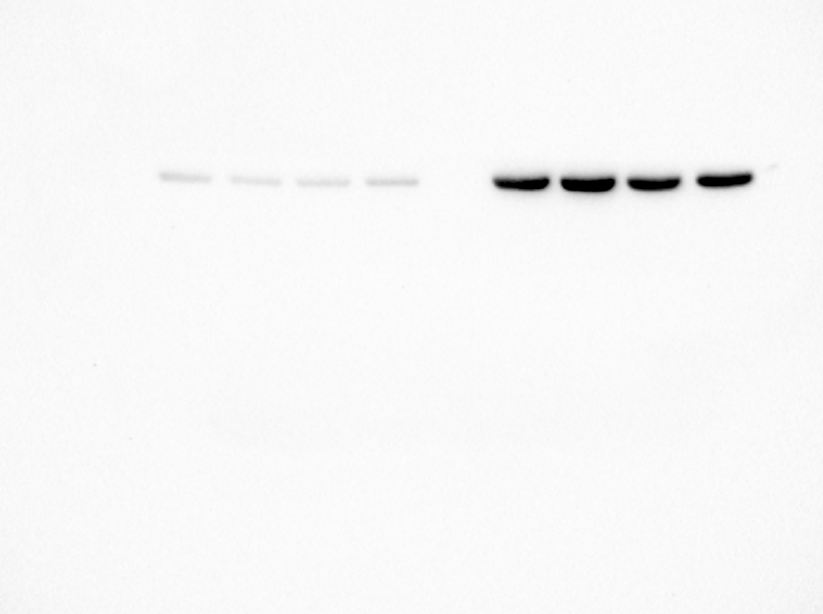


lamin-B


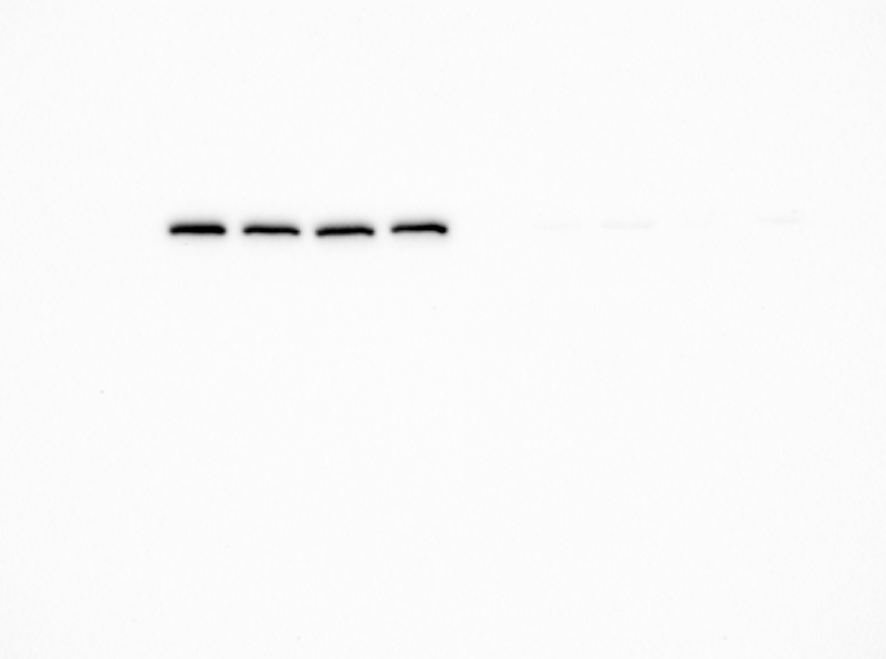


**Fig. 4.B**

NRF2


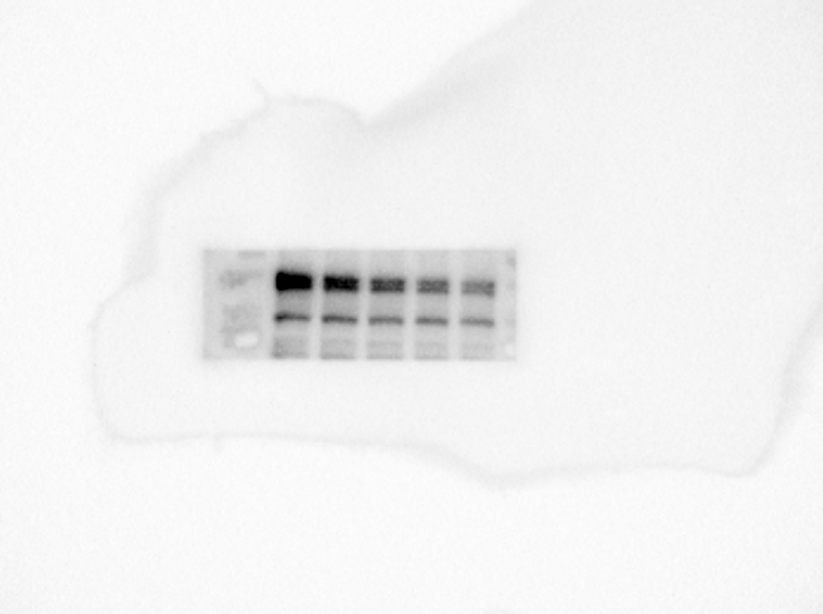


β-ACTIN


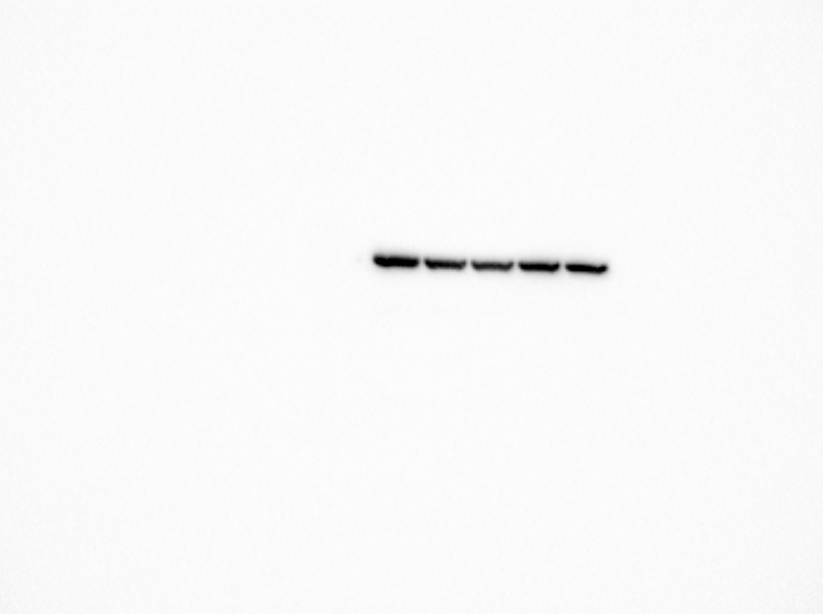


NRF2


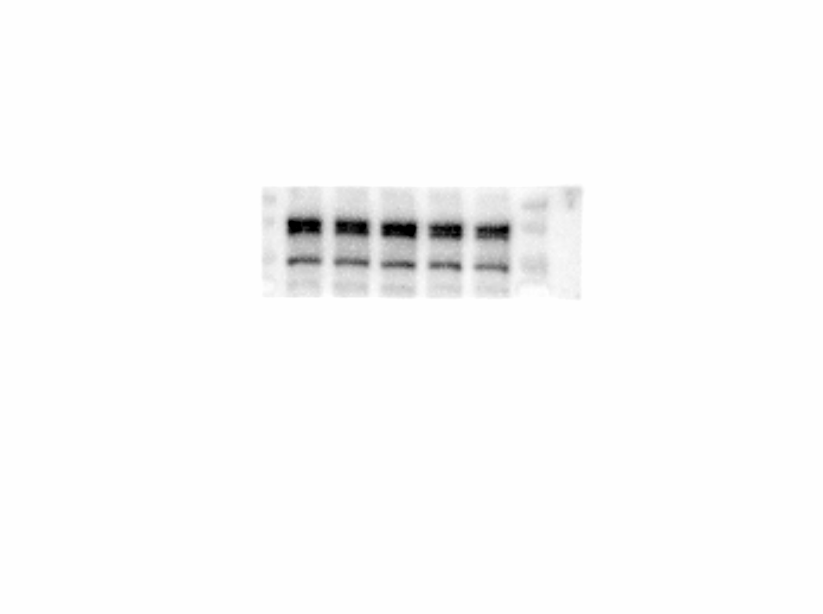


β-ACTIN


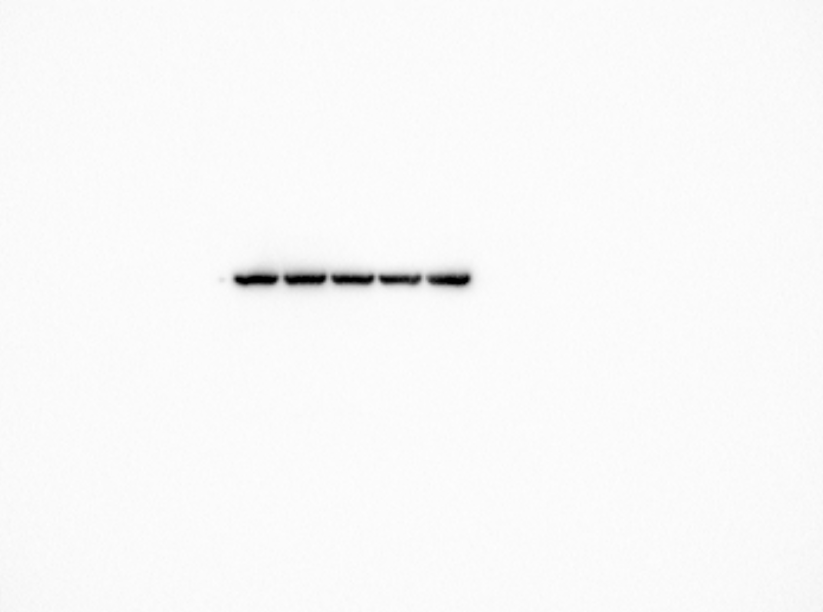


**Fig. 4.D**

BUI


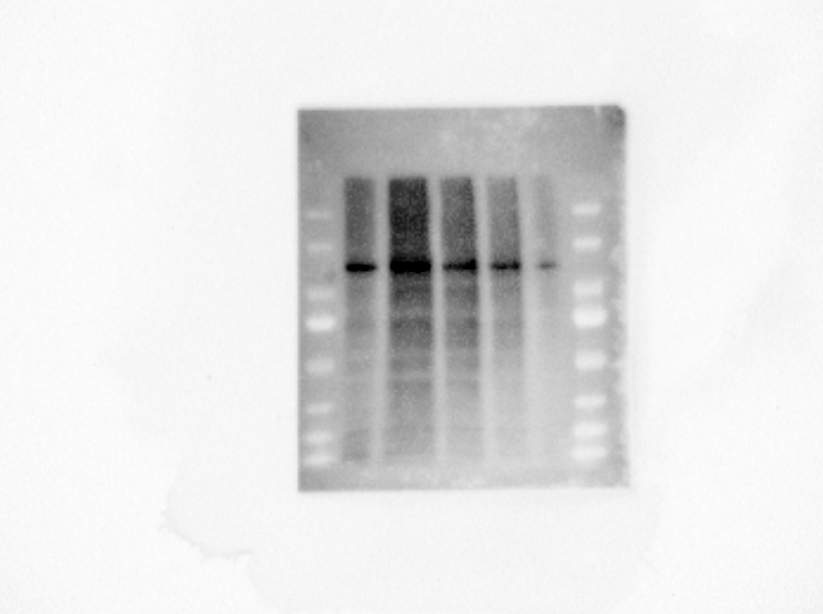


NRF2


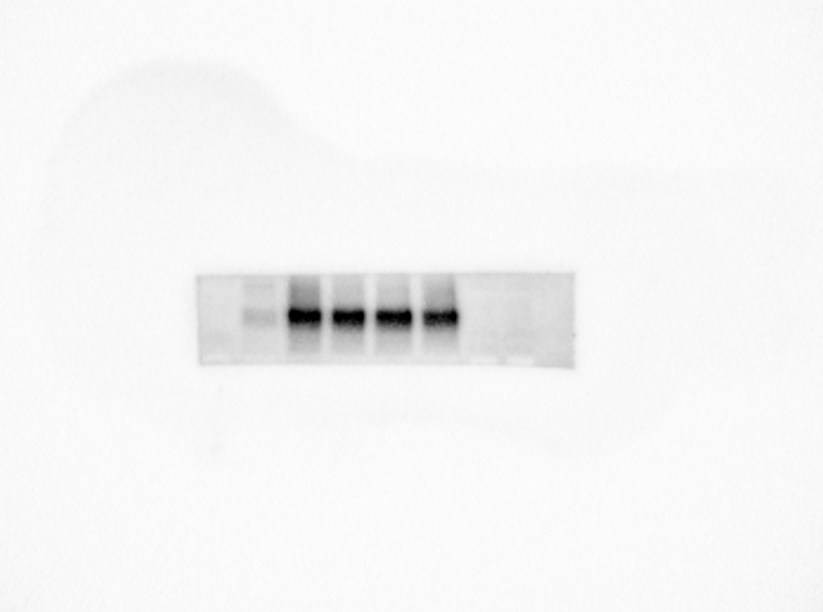


β-ACTIN


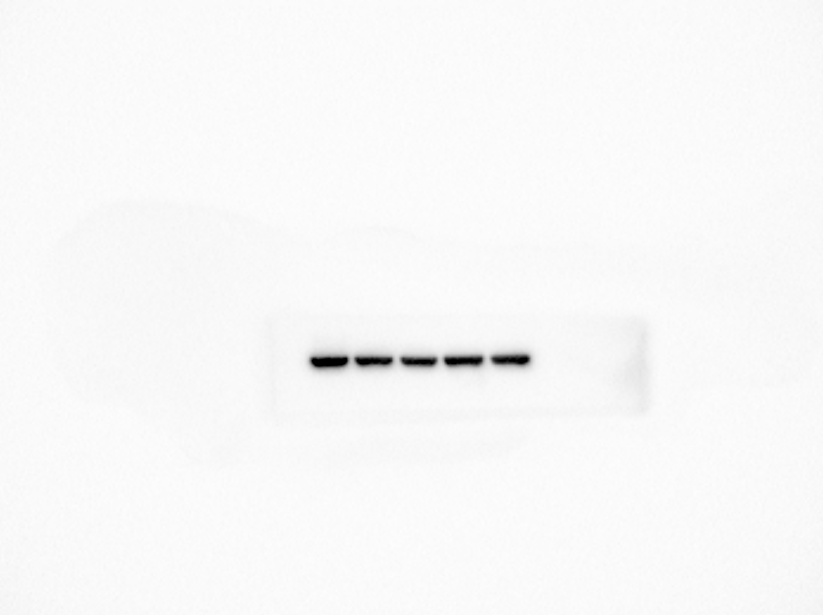


**Fig.4 F**

NRF2


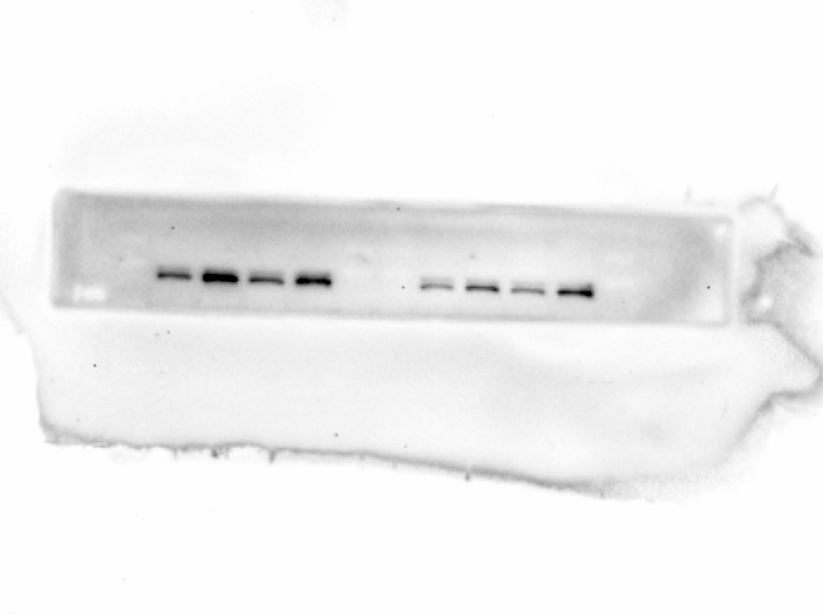


HO-1


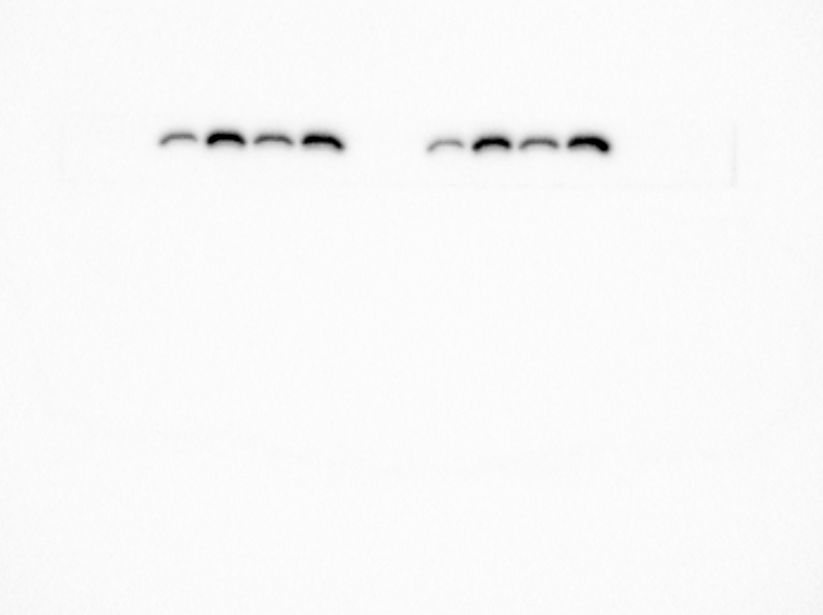


LC3B I/II


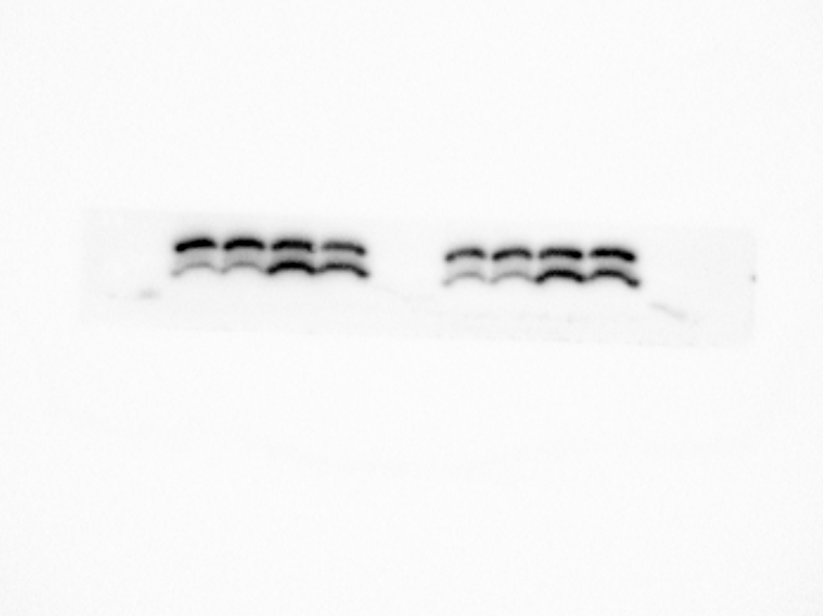


Β-actin


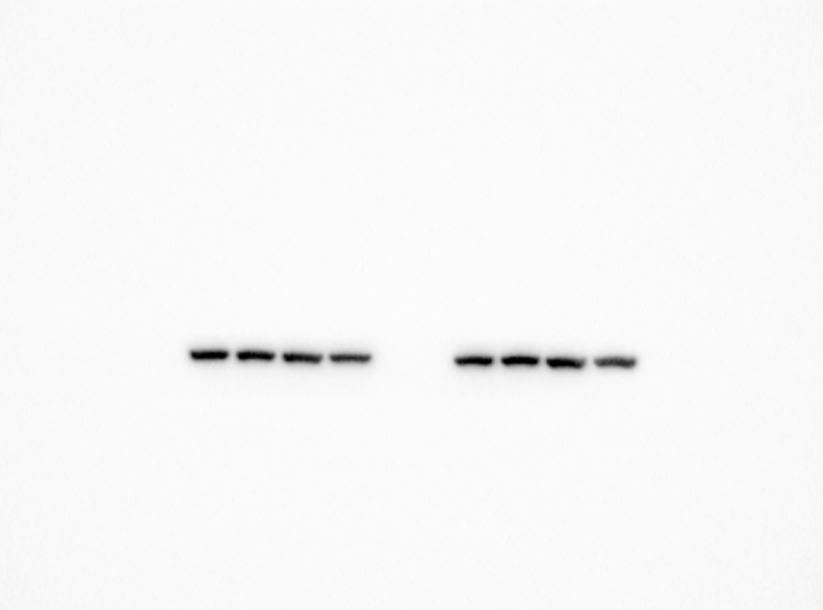


**Fig.4 E**

NRF2

**
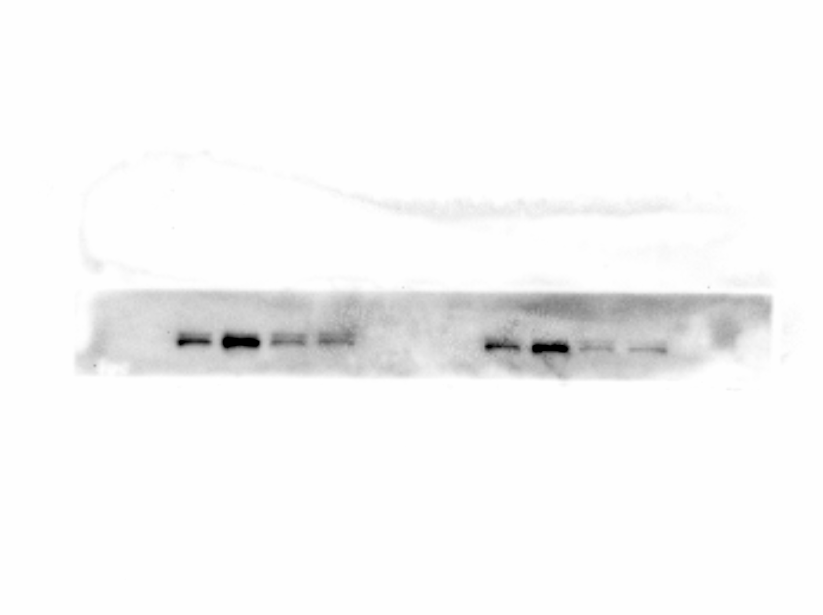
**

HO-1

**
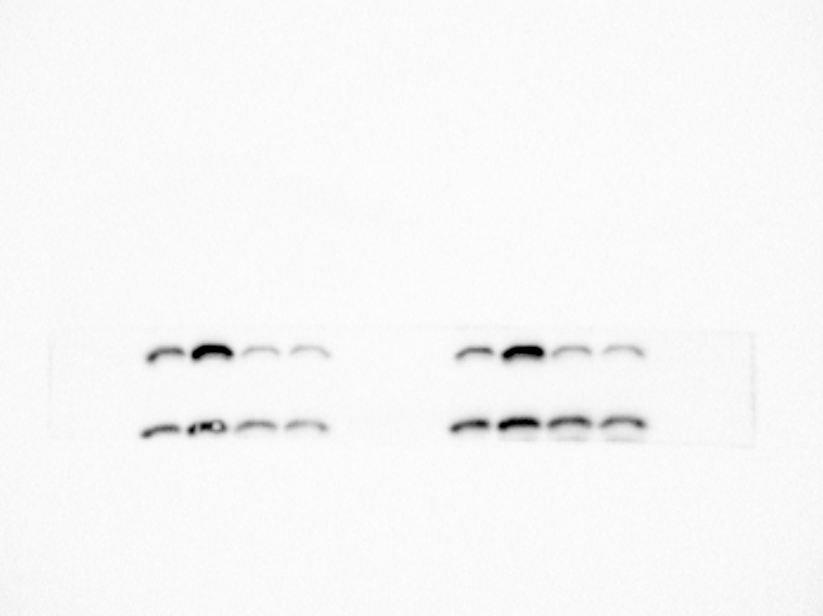
**

Β-actin


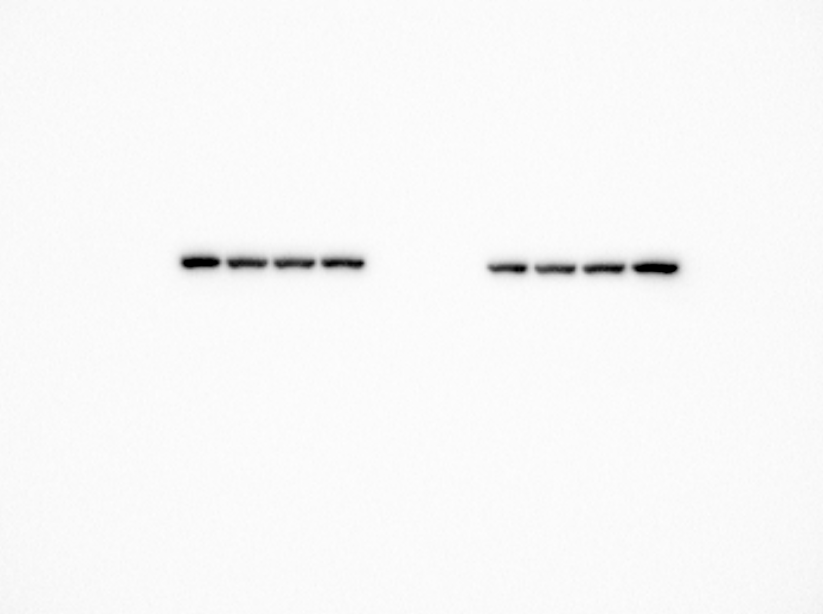


**Fig. 6. L**

γ-H2AX


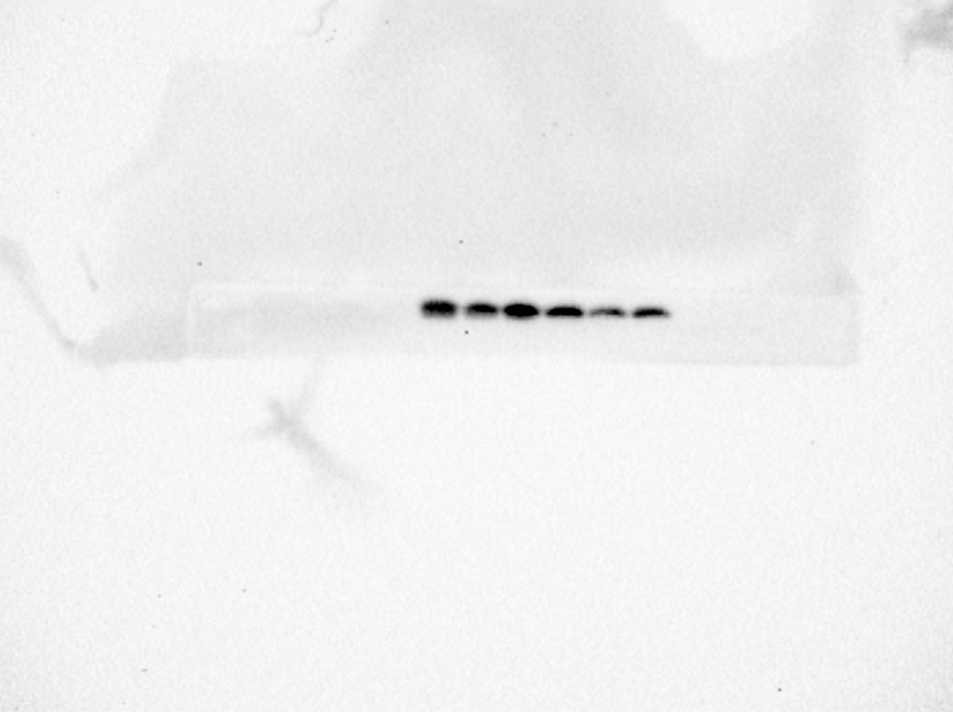


Clv-cas3


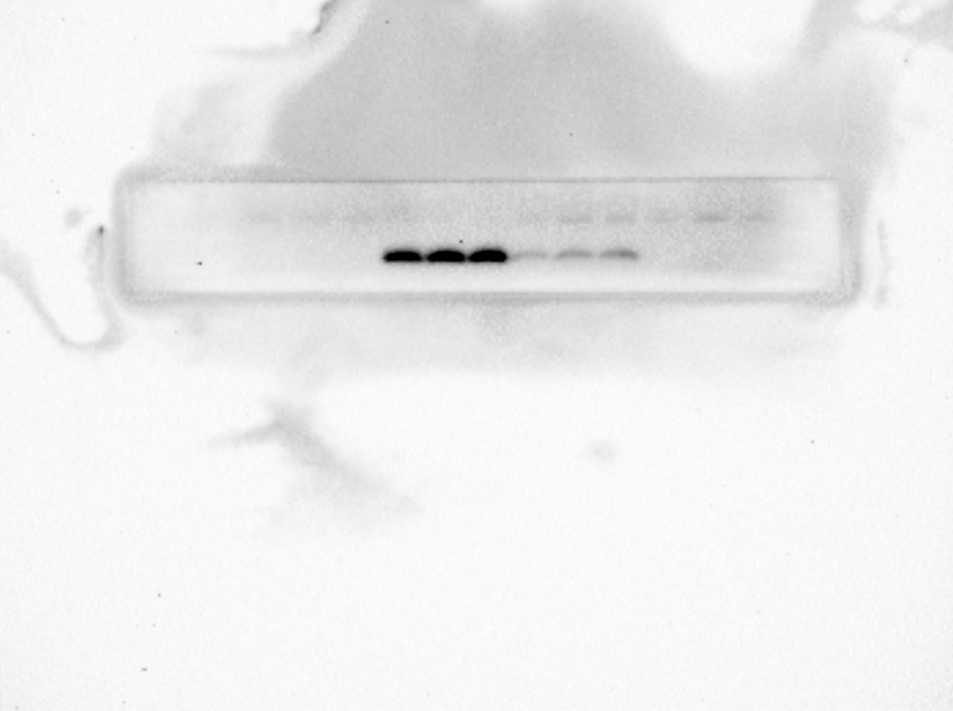


BAX


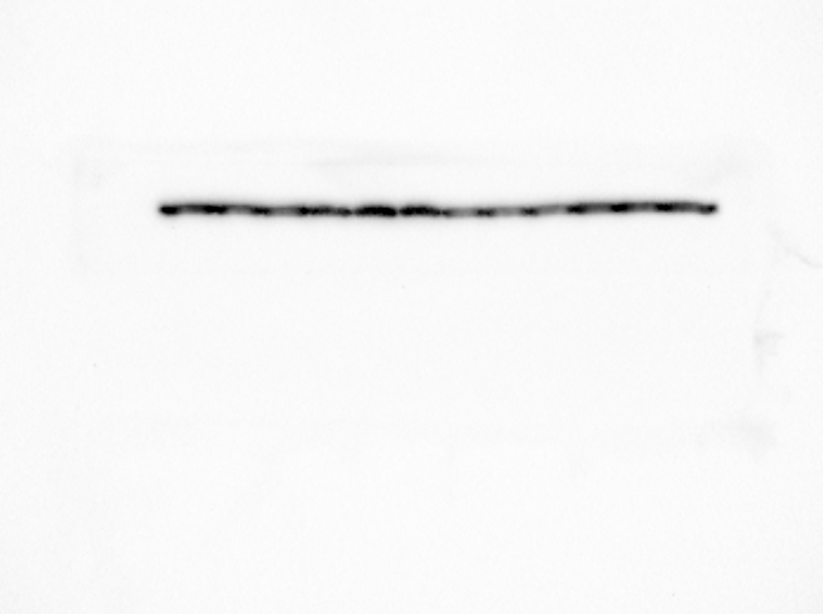


β-ACTIN


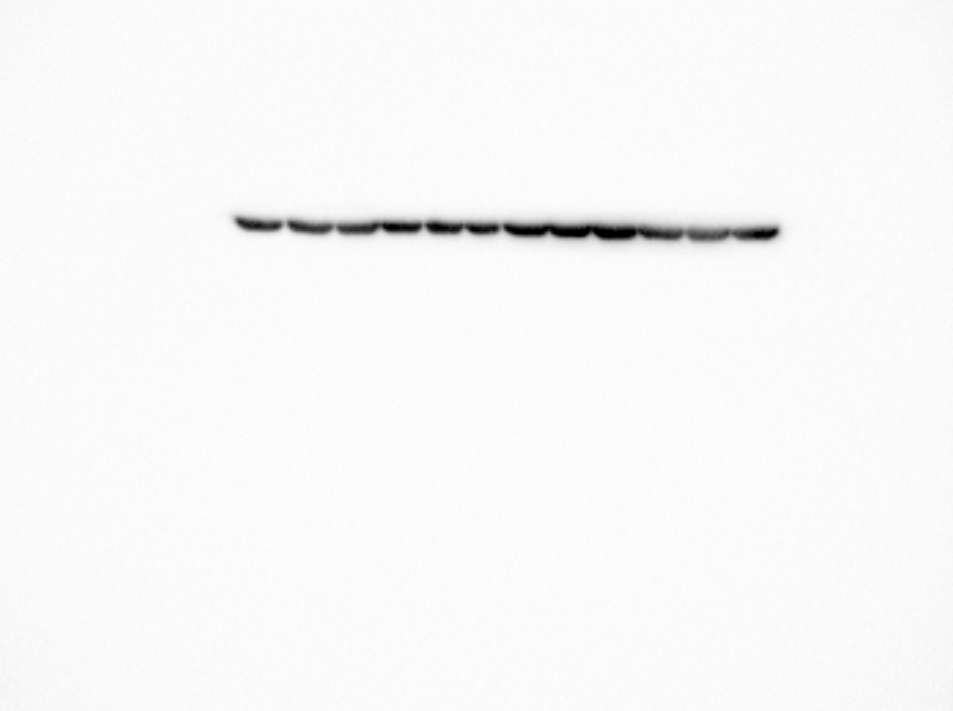


IL1β


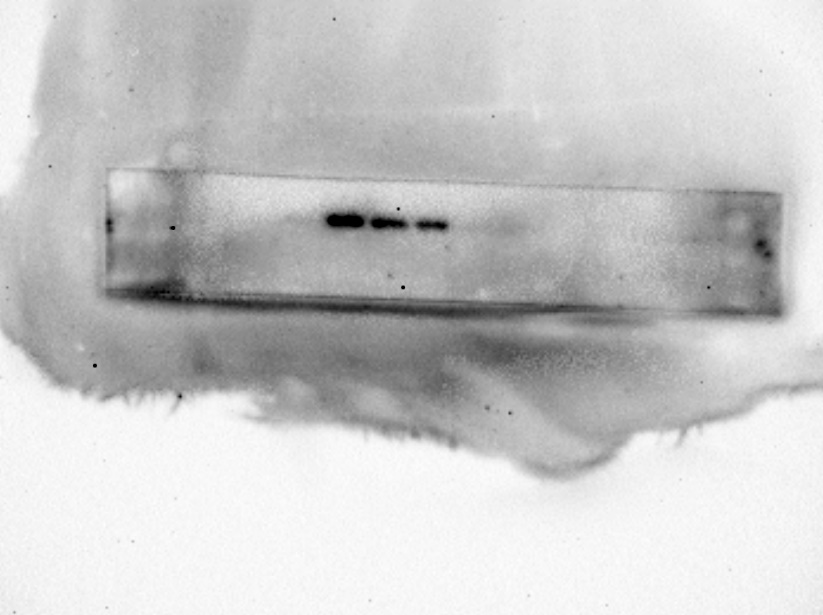


β-ACTIN


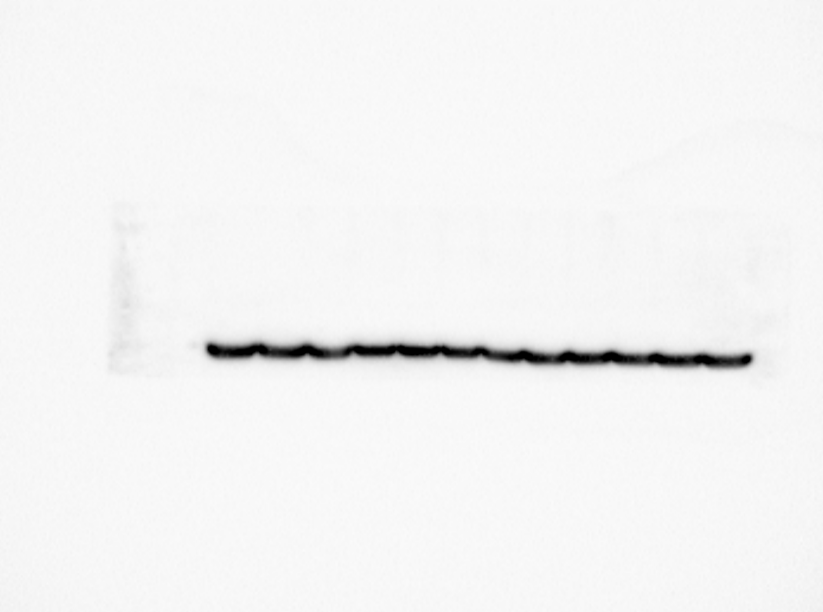


**Fig. 7. B**

NRF2


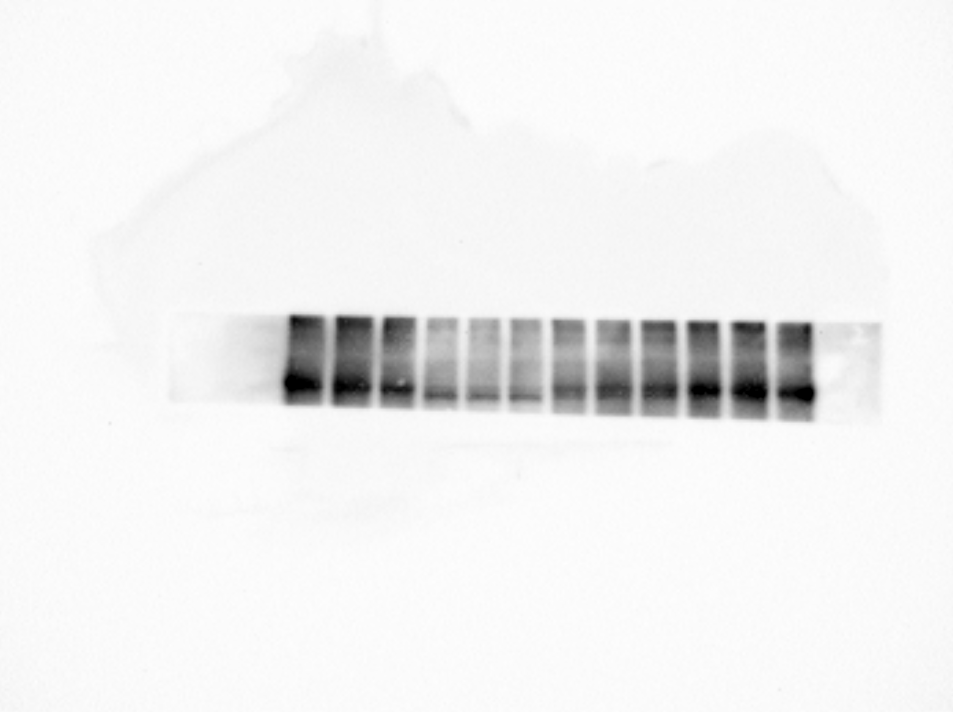


XCT


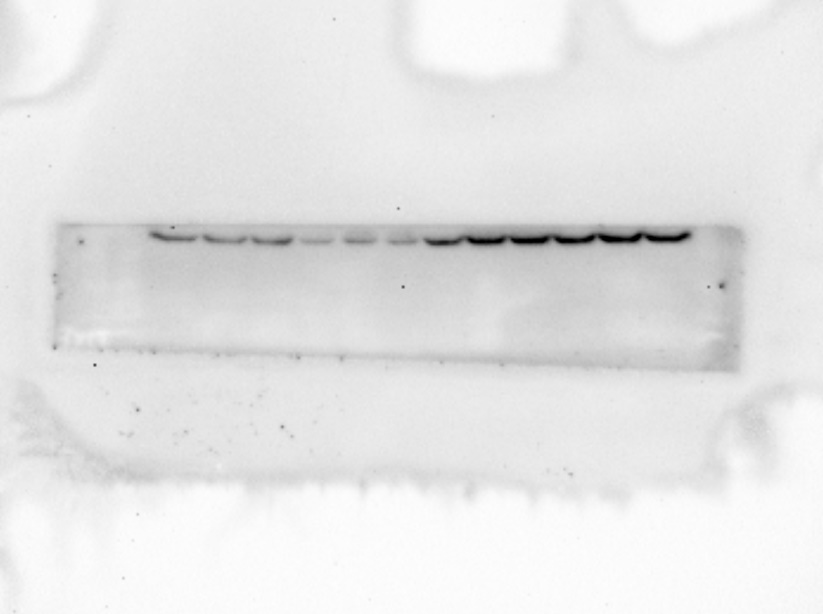


HO-1


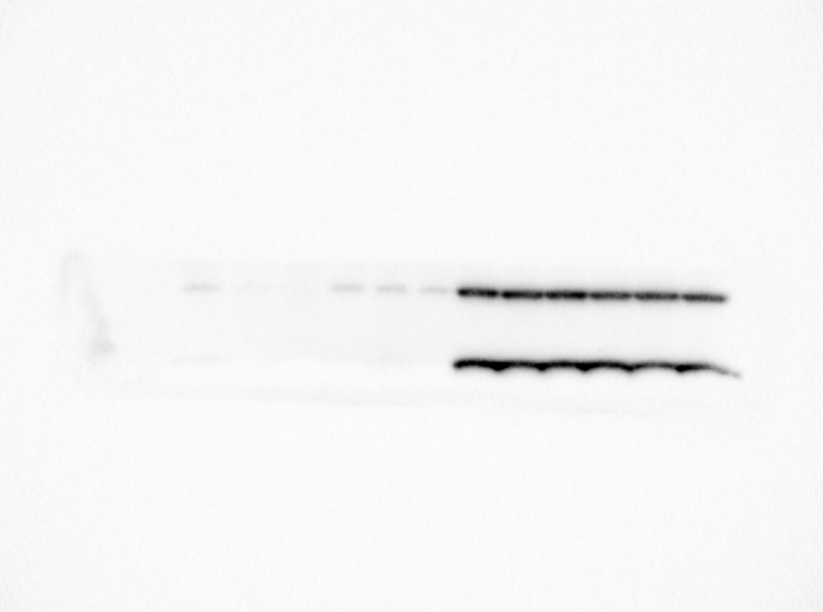


GCLM


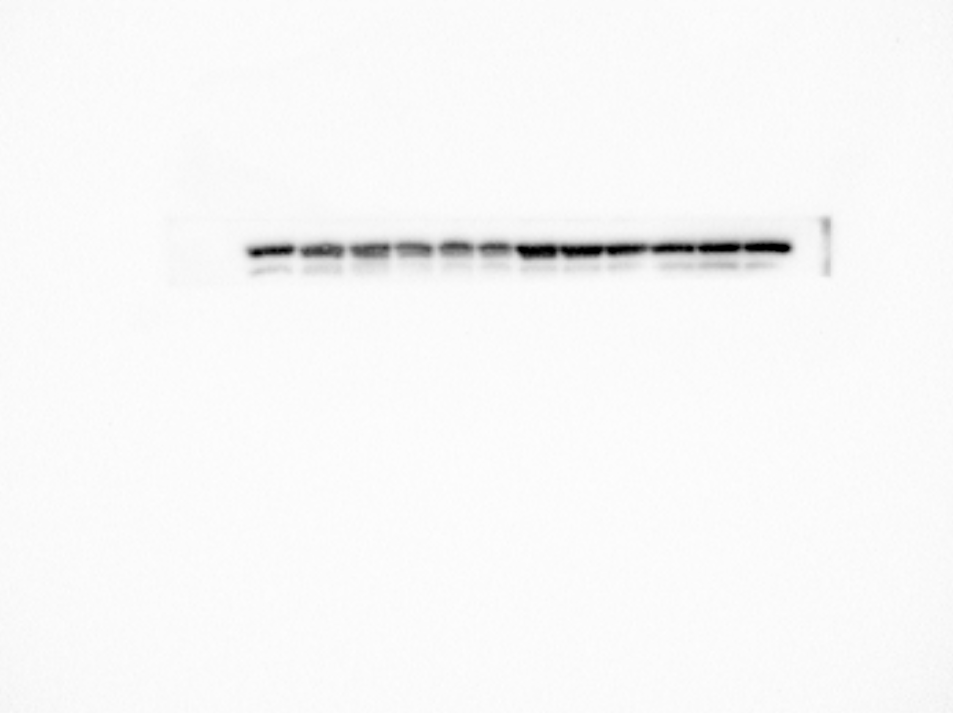


NQO1


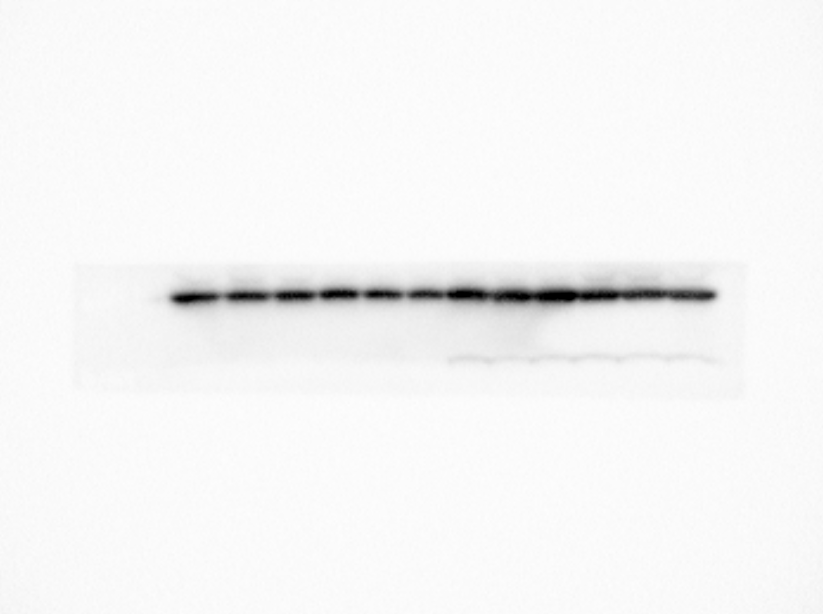


β-ACTIN


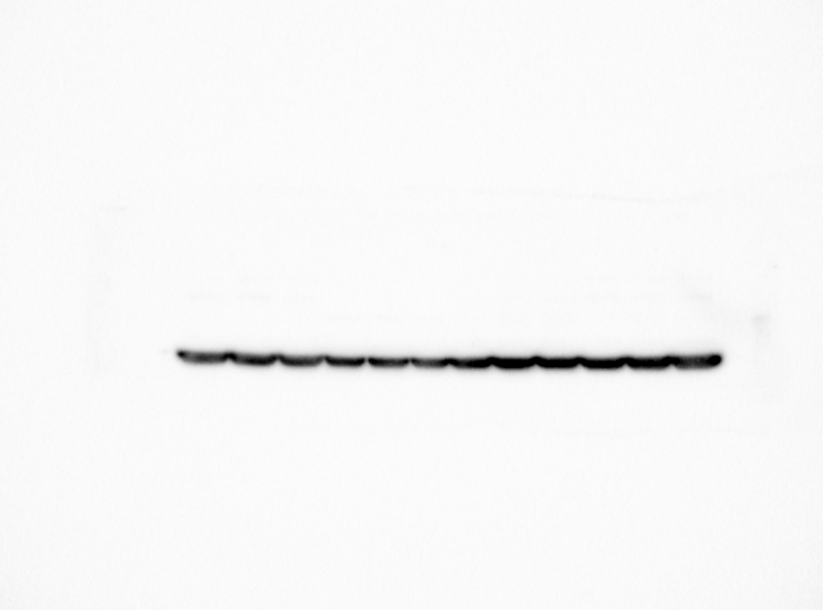


**Supplementary Fig. 3 A**

NRF2


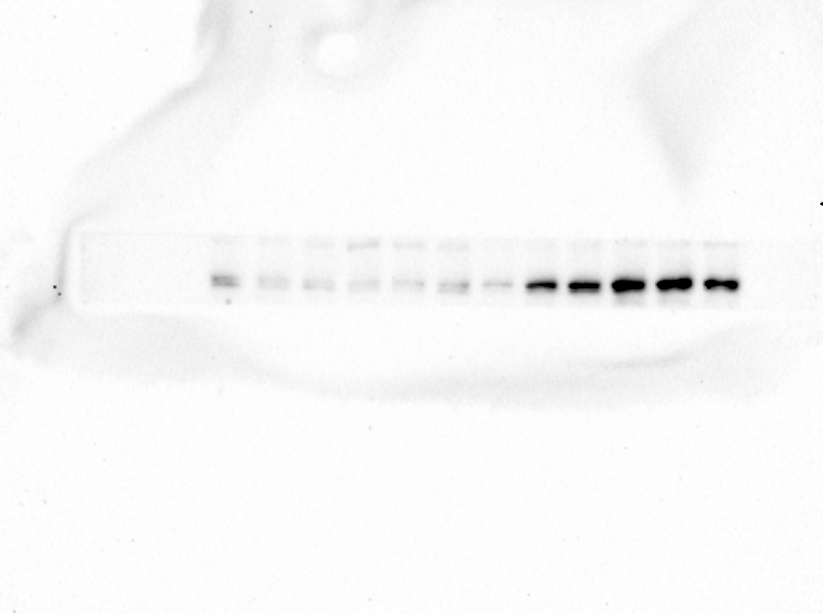


HO1


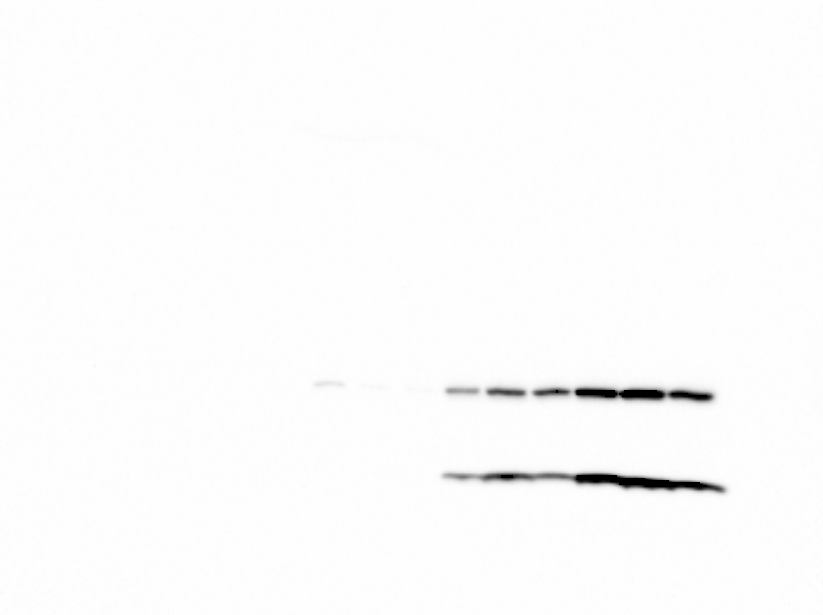


NQO1


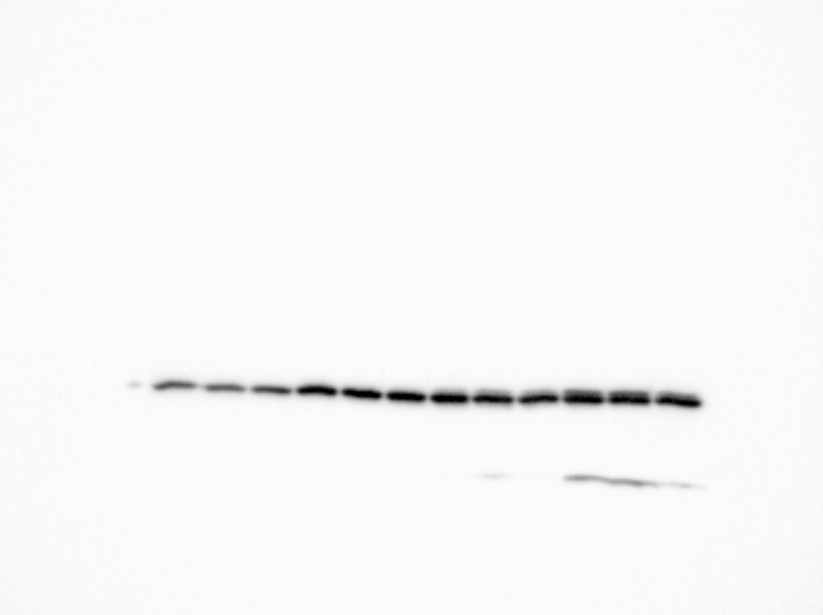


β-ACTIN


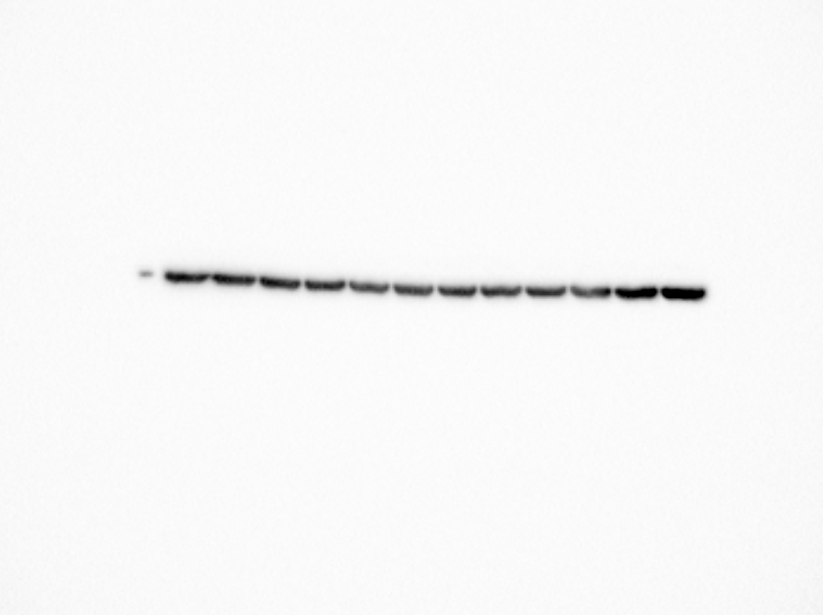


**Supplementary Fig. 3 A**

**Supplementary Fig. 3. C**

NRF2-brain


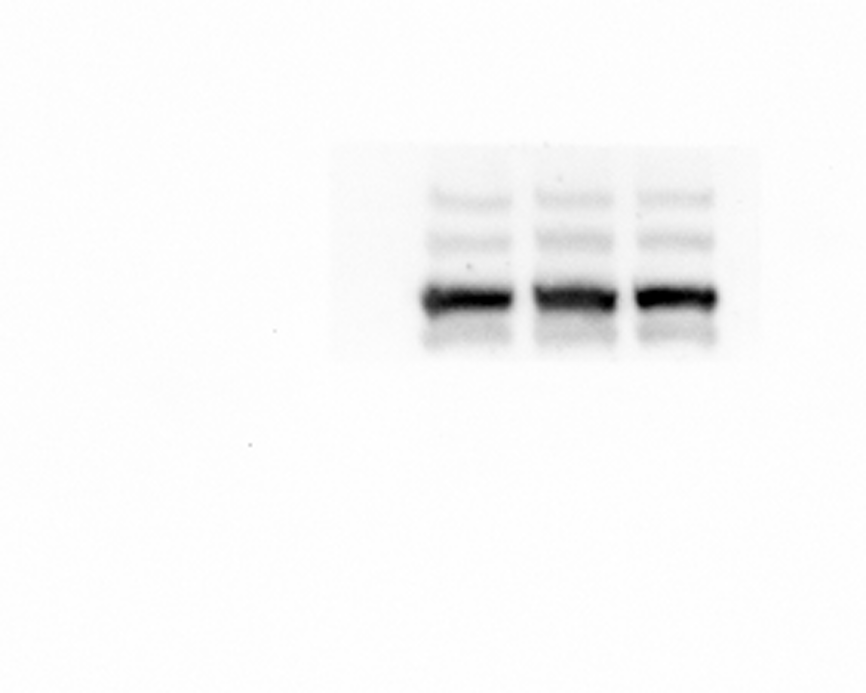


NRF2-lung


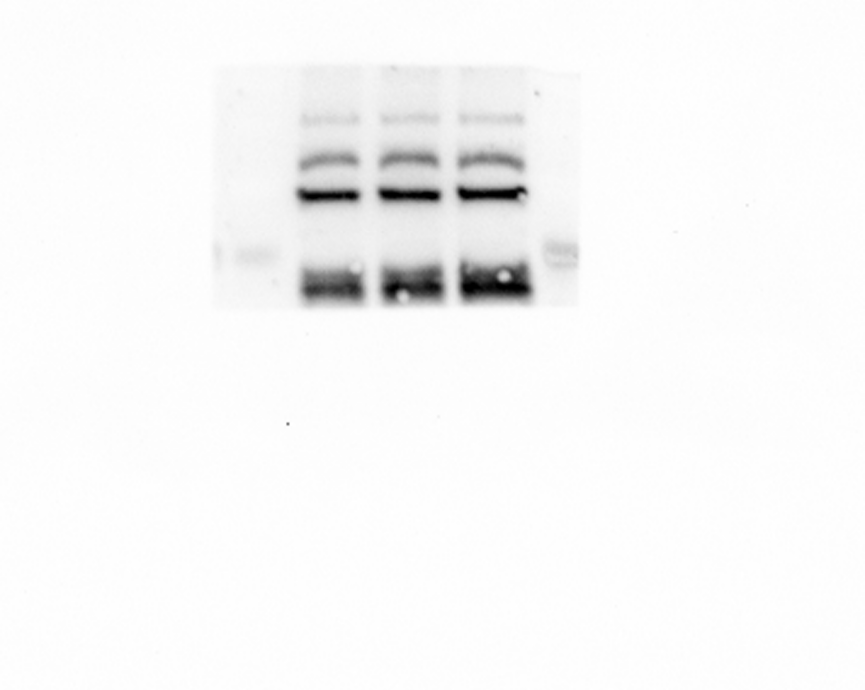


NRF2-heart


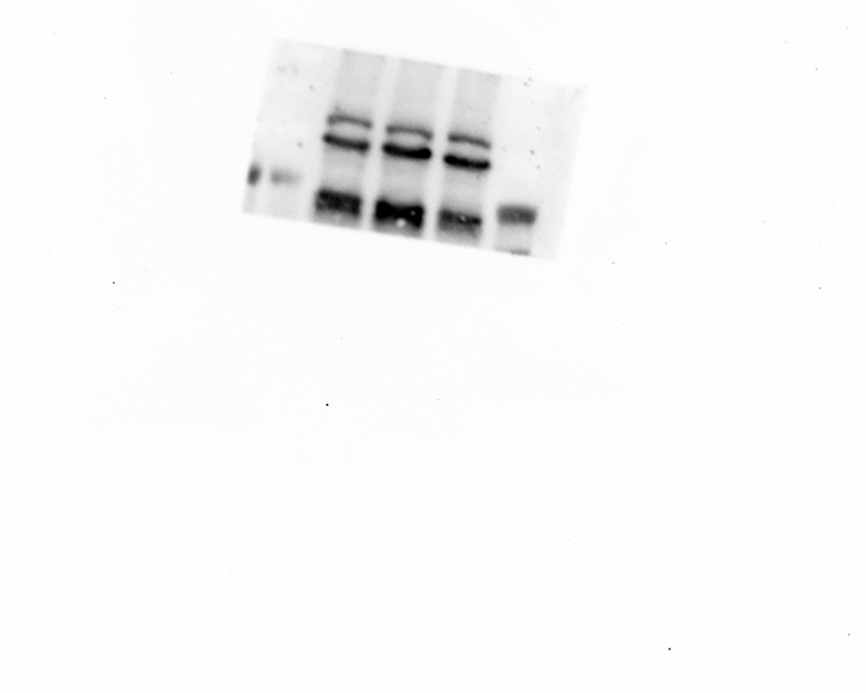


NRF2-kidney


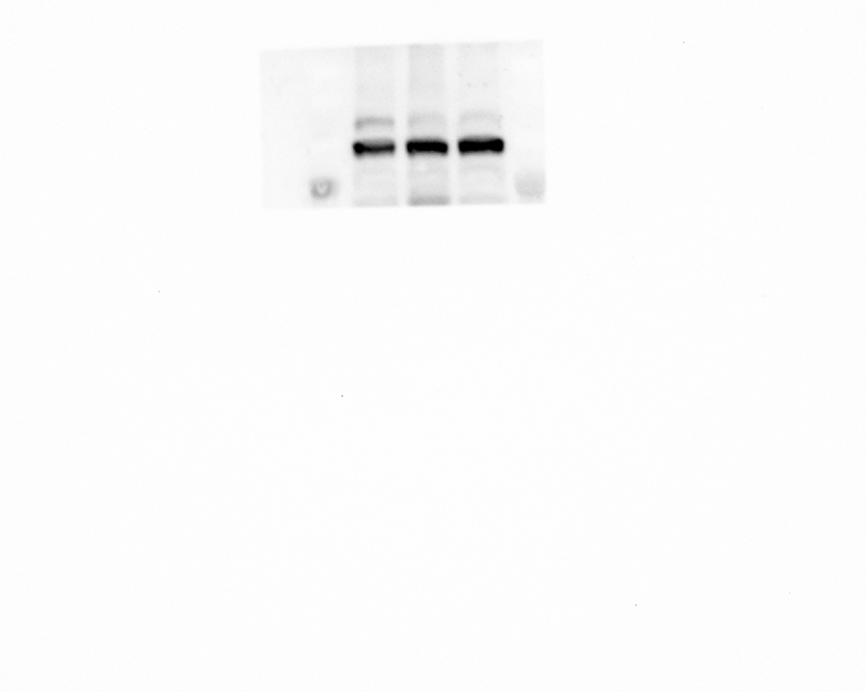


NRF2-liver


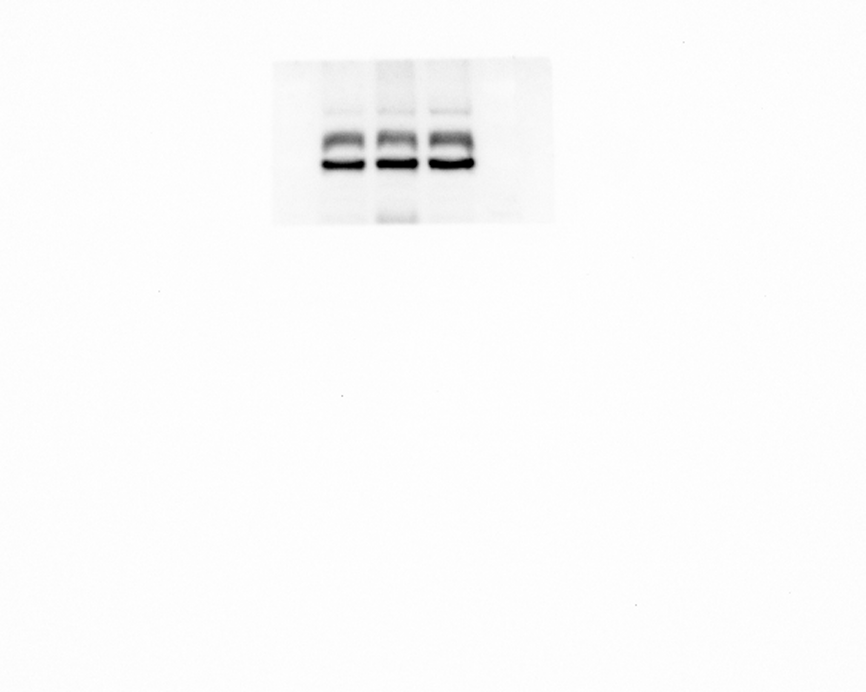


HO-1-brain


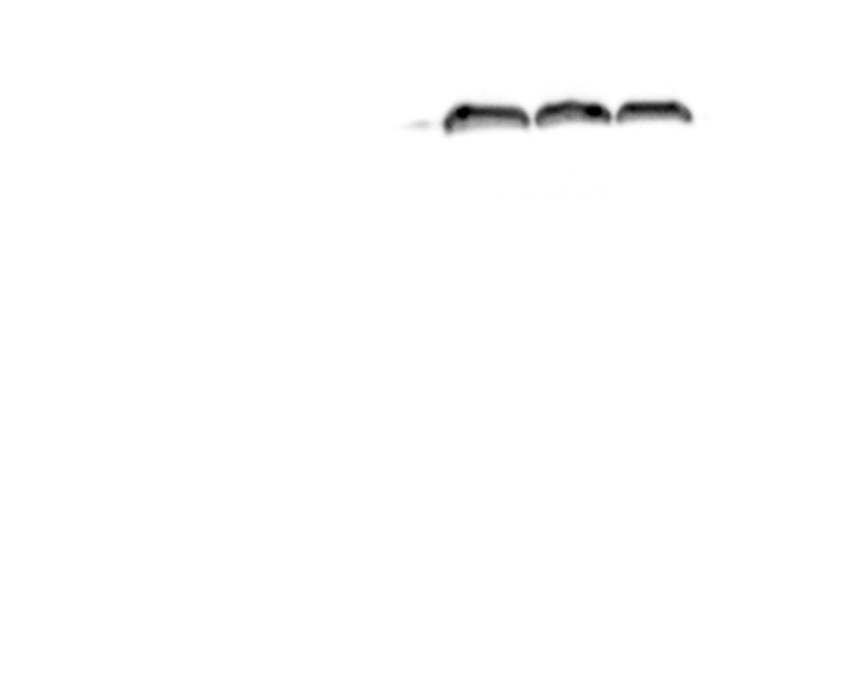


HO-1-lung


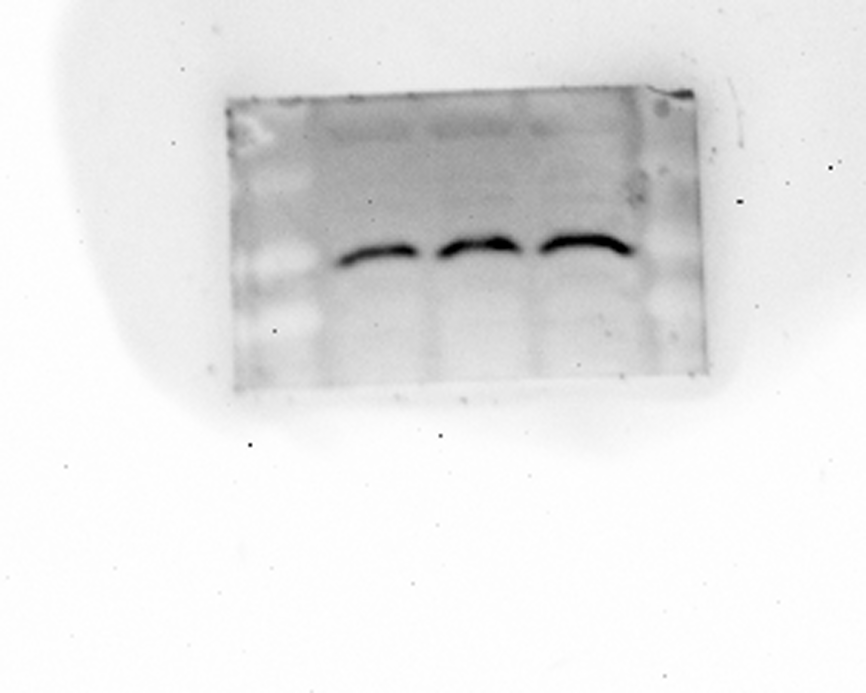


HO-1-heart


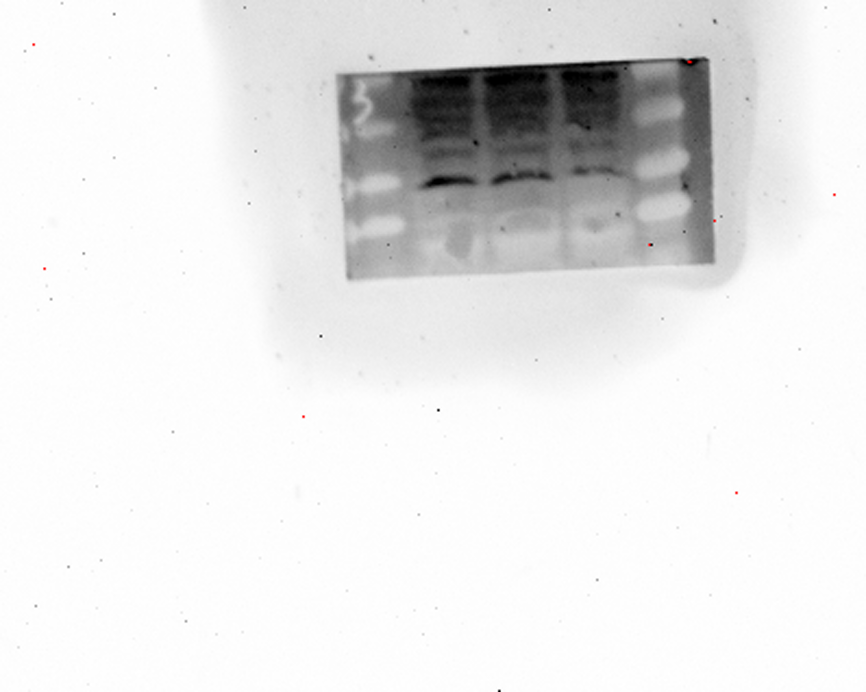


HO-1-kidney


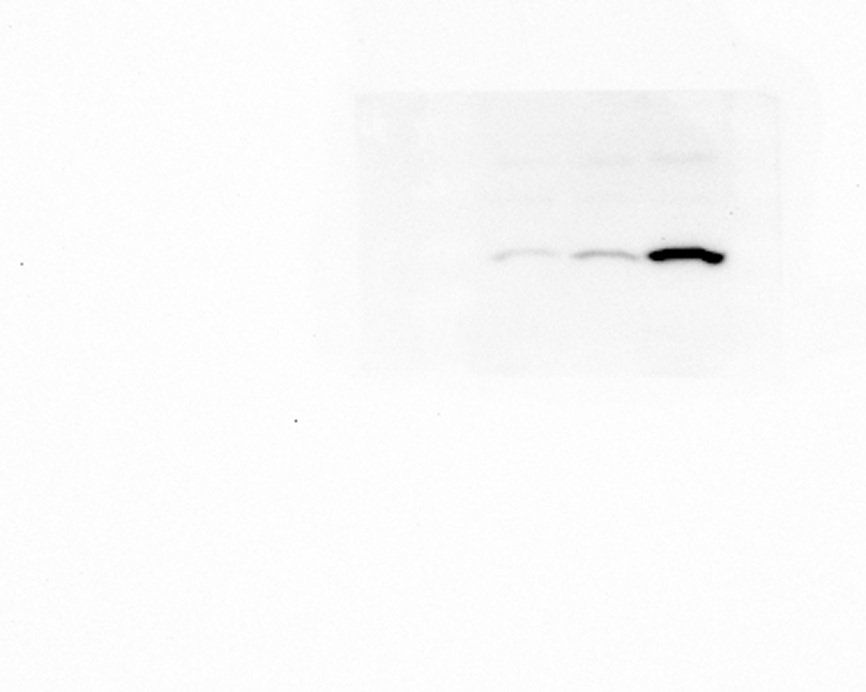


HO-1-liver


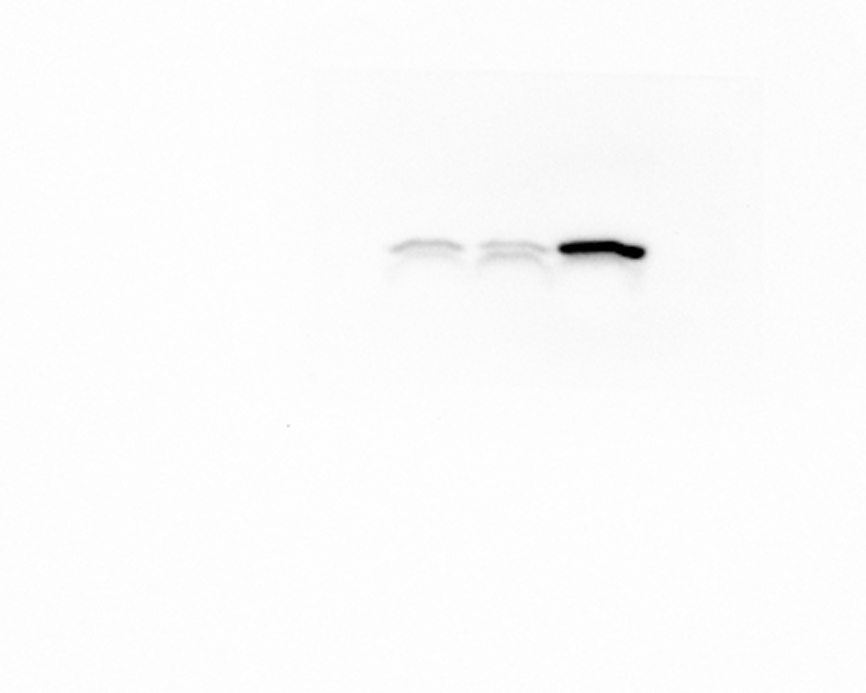


β-ACTIN-brain


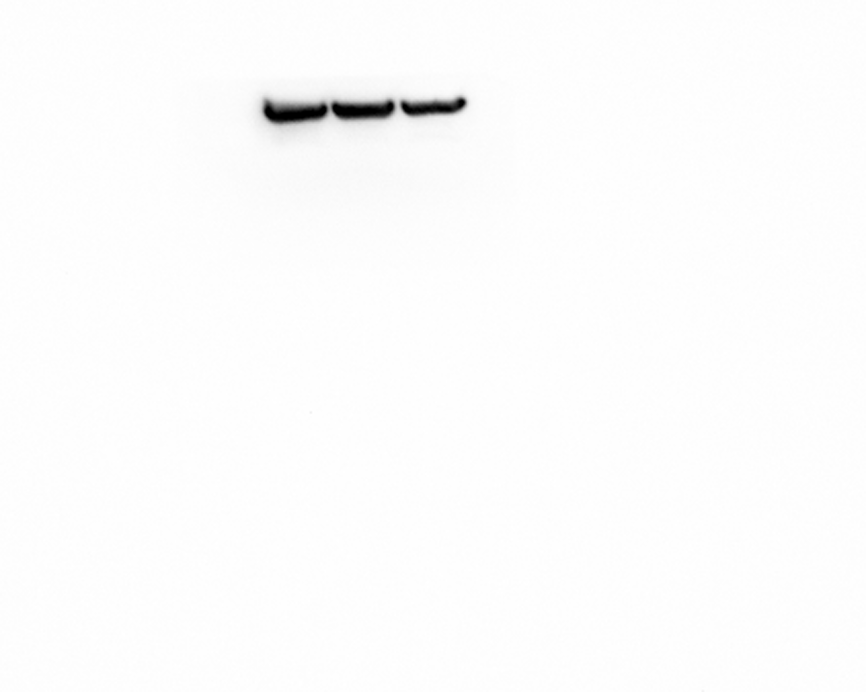


β-ACTIN-lung


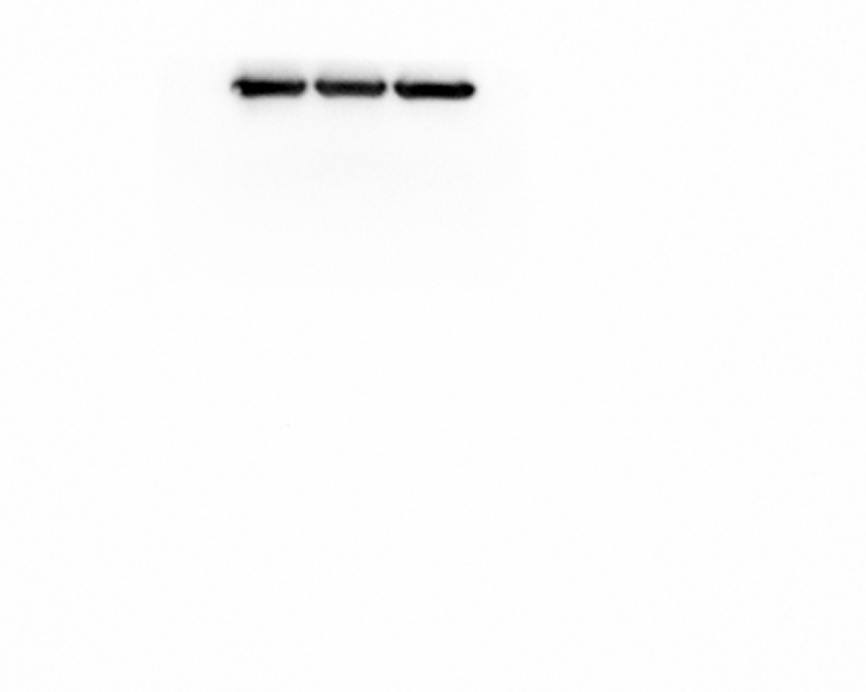


β-ACTIN-heart


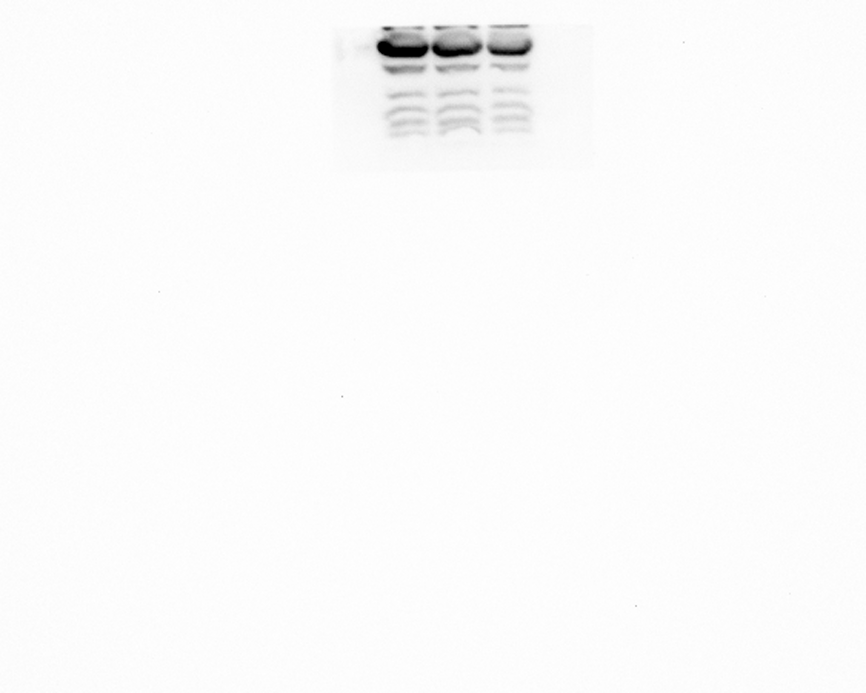


β-ACTIN-kidney


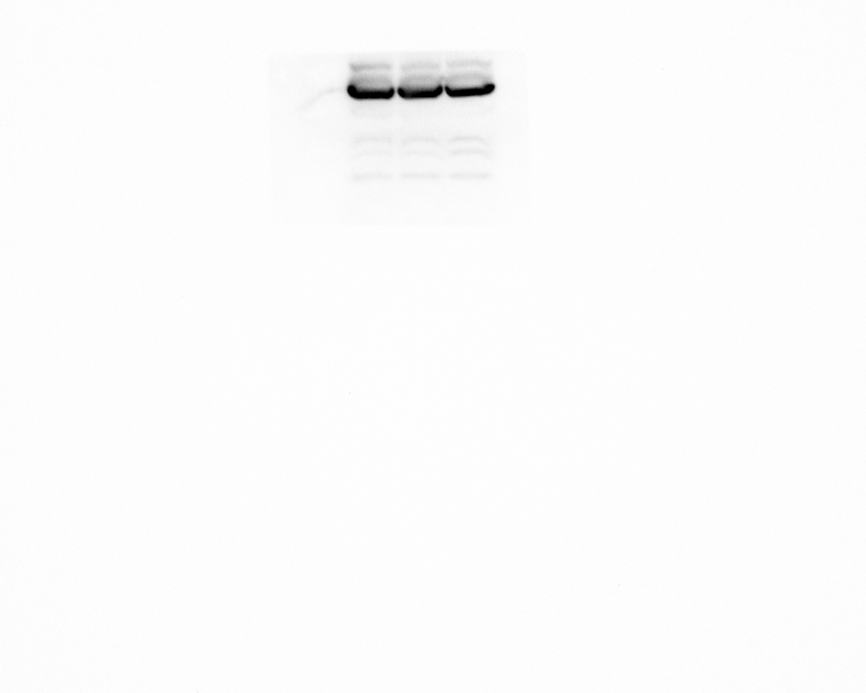


β-ACTIN-liver


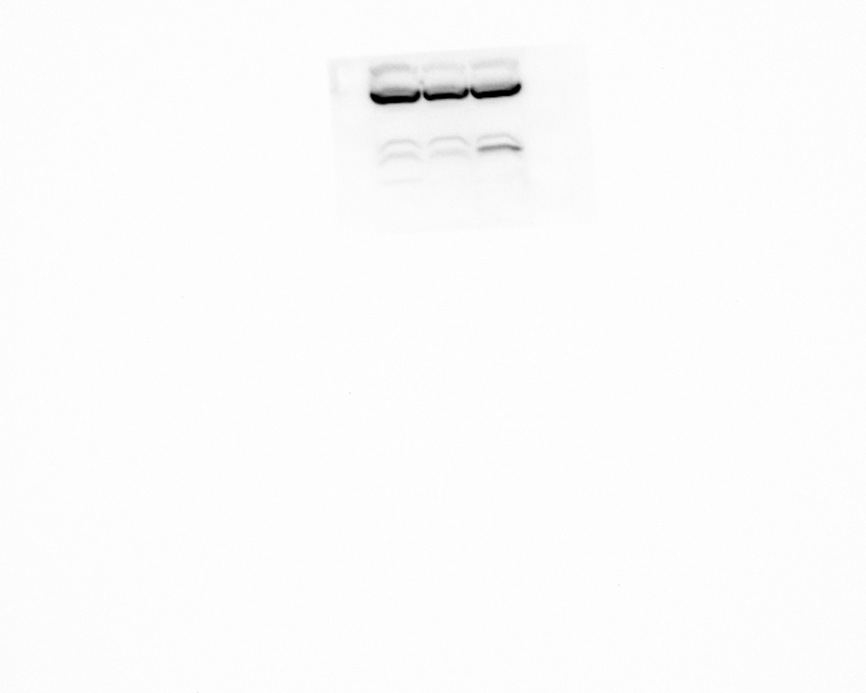

Supplement: Supplementary file 3 — Original Data File [file 41420_2023_1564_MOESM3_ESM.docx]
